# CF-DETR: a robust transformer-based framework for small-scale chili flower detection in industrial chili production systems

**DOI:** 10.3389/fpls.2026.1824412

**Published:** 2026-05-07

**Authors:** Minqiu Kuang, Xiaojian Li, Bei Wu, Dawei Liu, Yang Xiang, Feng Liu, Xiangjun Zou, Fangping Xie, Yuxuan Zhang, Xu Li

**Affiliations:** 1 College of Electrical and Mechanical Engineering, Hunan Agricultural University, Changsha, China; 2 Foshan-Zhongke Innovation Research Institute of Intelligent Agriculture and Robotics, Foshan, China; 3 Hunan Provincial Key Laboratory of Intelligent Agricultural Machinery Equipment, Changsha, China; 4 College of Horticulture, Hunan Agricultural University, Changsha, China; 5 College of Intelligent Science and Engineering, Beijing University of Agriculture, Beijing, China; 6 Department of Computer and Electrical Engineering, Mid Sweden University, Sundsvall, Sweden

**Keywords:** deep learning, flower detection, industrial chili pepper, precision horticulture, reproductive phenology

## Abstract

Chili pepper (Capsicum spp.) is a high-value industrial horticultural crop widely utilized in food processing, pharmaceuticals, and natural pigment production. Accurate monitoring of flowering is critical for yield formation, pollination management, and early-stage production forecasting in industrial chili production systems. However, in greenhouse environments, chili flowers typically exhibit small object scale and are affected by issues such as lighting variations and occlusion, which pose significant challenges for reliable visual detection. These factors often result in missed detections and unstable performance in practical phenological monitoring tasks. To address these challenges, this study proposes CF-DETR, a robust transformer-based framework for small-scale chili flower detection. Built upon the RT-DETR architecture, the proposed method introduces an efficiency-optimized FasterNet backbone to enhance fine-grained feature extraction for small targets while maintaining computational efficiency. In addition, a dynamic upsampling mechanism is incorporated to preserve structural details during feature reconstruction, and a Bidirectional Multi-scale Attention Feature Pyramid Network (BiMAFPN) is designed to strengthen cross-scale feature interaction under complex greenhouse backgrounds and occlusion conditions. Experiments conducted on a self-constructed greenhouse dataset demonstrate that CF-DETR achieves a Precision of 94.1%, mAP50 of 83.5%, and mAP50–95 of 64.5%, outperforming the baseline RT-DETR-r18 model. Furthermore, deployment on an NVIDIA Jetson AGX Orin platform achieves real-time inference at 30.65 FPS, validating its practical applicability in edge-enabled agricultural systems. The proposed framework provides a reliable visual sensing solution for small-scale phenology monitoring, enabling intelligent pollination management, early yield prediction, and data-driven decision-making in industrial chili production. This work contributes to the advancement of precision horticulture and the digital transformation of industrial crop production systems.

## 1 Introduction

Agricultural industrial crops constitute a critical pillar of global agro-processing economies, providing essential raw materials for food manufacturing, pharmaceuticals, cosmetics, nutraceuticals, and bio-based products. Among these, chili pepper (Capsicum spp.) represents one of the most economically significant horticultural industrial crops worldwide Barik et al. (2022). Beyond its role as a fresh vegetable, chili serves as a high-value industrial feedstock. Capsaicinoids extracted from chili are extensively applied in pharmaceutical analgesics and anti-inflammatory formulations, as well as in cosmetic products designed for topical stimulation and metabolic activation. Natural carotenoid pigments such as capsanthin and capsorubin are widely utilized in food coloring and functional health products Govindarajan and Salzer (1985). In addition, processed chili derivatives, including powders, oleoresins, sauces, and extracts, form an important segment of agro-industrial value chains.

China, as the world’s largest producer and consumer of chili peppers Kuang et al. (2026a); Tang et al. (2023a); Zhou et al. (2025), places strategic importance on the high-quality development of this industry to ensure vegetable supply security and promote agricultural economic growth. Consequently, genetic improvement of germplasm resources, yield trait prediction, and reproductive-stage monitoring have remained central topics in agronomic and breeding research Ollerton et al. (2014). From an industrial perspective, production stability directly influences downstream processing capacity, supply chain reliability, and market price volatility. Therefore, improving reproductive efficiency and ensuring stable fruit set are not only agronomic objectives but also industrial imperatives.

Flowering represents the most critical phenological stage in chili production systems. The number, spatial distribution, and physiological condition of flowers directly determine fruit set rates and final yield output. Meanwhile, with the evolution of global agricultural ecosystems, the conservation of pollinators and the monitoring of crop pollination efficiency have attracted increasing attention Aquino et al. (2018). In modern precision agriculture and smart greenhouse Internet-of-Things (IoT) systems, flowering monitoring serves not only as a key indicator for evaluating crop vigor and pollination quality but also as a critical basis for early-stage yield prediction and data-driven agronomic decision-making Zhang et al. (2026b); Tang et al. (2023b); Chen et al. (2023). Compared with delayed statistics derived from fruit ripening stages, high-throughput phenological analysis of flowering using machine vision enables real-time sensing and intelligent control within agricultural IoT monitoring frameworks, significantly improving the timeliness and reliability of crop management Lu et al. (2026); Zhang et al. (2026a), Zhang et al. (2026d); Liang et al, 2025, Liang et al, 2026); Zhang et al. (2026c); Xue et al. (2026); Mendez et al. (2025); Shaowei et al. (2026).

With the rapid advancement of deep learning, visual perception technologies have achieved remarkable progress across multiple domains, including fruit and vegetable image analysis Ma et al. (2026); Kuang et al. (2026c); Cao et al. (2025); Zhang et al. (2026a; 2026d). Li et al. Li et al. (2024) and Li et al. Li et al. (2021) conducted detection studies on chili fruits and green peppers in natural environments, validating the capability of convolutional neural networks to handle complex agricultural scenes and providing methodological references for flowering monitoring. For crops with relatively large flowers and distinct morphological structures, existing detection techniques have matured considerably. Wu et al. Wu et al. (2020) and Shang et al. Shang et al. (2023) achieved real-time apple flower detection via lightweight backbone optimization. Sun et al. Sun et al. (2021) and Tian et al. Tian et al. (2020) further employed semantic segmentation and improved Mask R-CNN models to realize fine-grained growth-stage recognition and differentiation between central and lateral flowers.

Beyond orchard crops, similar visual sensing paradigms have been extended to lotus counting Lyu et al. (2024), grapevine inflorescence monitoring Li et al. (2018); Palacios et al. (2020), greenhouse tomato flowering detection under occlusion Zhao et al. (2020); Houtman et al. (2021), and UAV-based density estimation frameworks Zhang et al. (2022). For dense flower perception tasks, researchers have explored multi-scale fusion and attention mechanisms. Guo et al. Guo et al (2022b) , Guo et al (2022a) improved YOLO-based architectures for kiwifruit bud detection, while Li et al. Li et al. (2025) introduced Transformer-based hybrid networks to address illumination and occlusion disturbances. Ye et al. Ye et al. (2023) proposed a Polyphyletic loss to alleviate bounding-box repulsion in litchi flower detection, and Bai et al. Bai et al. (2024) as well as Xu et al. Xu et al. (2026) demonstrated the advantages of Transformer-based attention mechanisms for strawberry reproductive monitoring in greenhouse environments.

Despite these advances, robust phenological sensing of chili flowers remains technically challenging. Chili flowers are small, densely clustered, frequently occluded by foliage, and exhibit subtle color contrast with surrounding leaves. In greenhouse environments characterized by complex backgrounds and dynamic illumination, these factors collectively result in unstable detection performance and high missed-detection rates. Previous efforts such as Wang et al. Wang and Zhang (2025) and Kuang et al. Kuang et al. (2026b), Kuang et al, 2025) improved YOLO variants through attention modules and transfer learning strategies, achieving competitive accuracy with reduced computational overhead. However, these paradigms fundamentally depend on Non-Maximum Suppression (NMS) in post-processing. In densely clustered flowering scenes, the high spatial overlap between adjacent targets frequently causes true positives to be erroneously suppressed, leading to systematic underestimation of flowering density. Additionally, lightweight CNN backbones typically possess limited receptive fields, making it difficult to capture subtle micro-textures of tiny flowers and rendering models vulnerable to lighting fluctuations and cluttered greenhouse backgrounds.

From an industrial-scale monitoring perspective, reliable small-object perception under dense, occluded, and unstructured greenhouse conditions remains a critical technological bottleneck. Addressing this challenge requires detection architectures capable of global context modeling, effective multi-scale feature fusion, and suppression-free target assignment mechanisms.

To overcome these limitations, this study proposes CF-DETR, a high-performance chili flowering detection framework built upon the RT-DETR architecture. By integrating efficient backbone redesign and enhanced multi-scale attention mechanisms, the proposed model achieves robust perception of densely clustered chili flowers while maintaining computational feasibility for edge deployment in greenhouse production systems. This work contributes to the digital transformation of industrial chili cultivation by enabling reliable reproductive-stage monitoring for intelligent pollination management, yield forecasting, and data-driven agro-industrial planning. The main contributions of this paper are summarized as follows:

- To address the challenge of balancing high-precision perception with computational feasibility in industrial greenhouse environments, a CF-DETR framework based on the RT-DETR architecture is proposed. By integrating the efficiency-optimized FasterNet as the backbone, the model significantly reduces computational redundancy while enhancing fine-grained spatial feature representation, providing a lightweight yet robust solution for edge-side monitoring of reproductive phenology;
- To overcome the severe information loss and feature ambiguity caused by the minute size, dense clustering, and frequent foliage occlusion of chili flowers, a Bidirectional Multi-scale Attention Feature Pyramid Network (BiMAFPN) and a dynamic upsampling mechanism are developed. These modules strengthen cross-scale feature interaction and preserve structural details during reconstruction, fundamentally improving detection robustness under unstructured field conditions;
- To bridge the gap between algorithmic research and stable agro-industrial supply chains, the practical applicability of the model is validated through deployment on the NVIDIA Jetson AGX Orin platform. Achieving a real-time inference speed of 30.65 FPS with high precision, the proposed system provides reliable visual support for intelligent pollination management and early-stage yield forecasting, thereby facilitating the digital transformation of industrial chili production.

The remainder of this paper is organized as follows: Section 1 provides a comprehensive review of the state-of-the-art in agricultural small object detection and visual perception. Section 2 outlines the materials and methodologies employed, with specific focus on the acquisition of experimental materials and the construction of the dataset. Section 3 offers a detailed description of the CF-DETR framework, systematically dissecting the FasterNet backbone and the BiMAFPN module, while also defining the experimental configuration and evaluation criteria. Section 4 presents the experimental validation and a comprehensive discussion. This section reports the results from ablation studies, comparative analyses, heatmap visualizations, and edge deployment tests, followed by an in-depth analysis of the findings from both quantitative and qualitative perspectives. Finally, Section 5 summarizes the key contributions of this study and suggests potential avenues for future investigation.

## 2 Materials and methods

### 2.1 Image acquisition and dataset preprocessing

**Figure 1:**
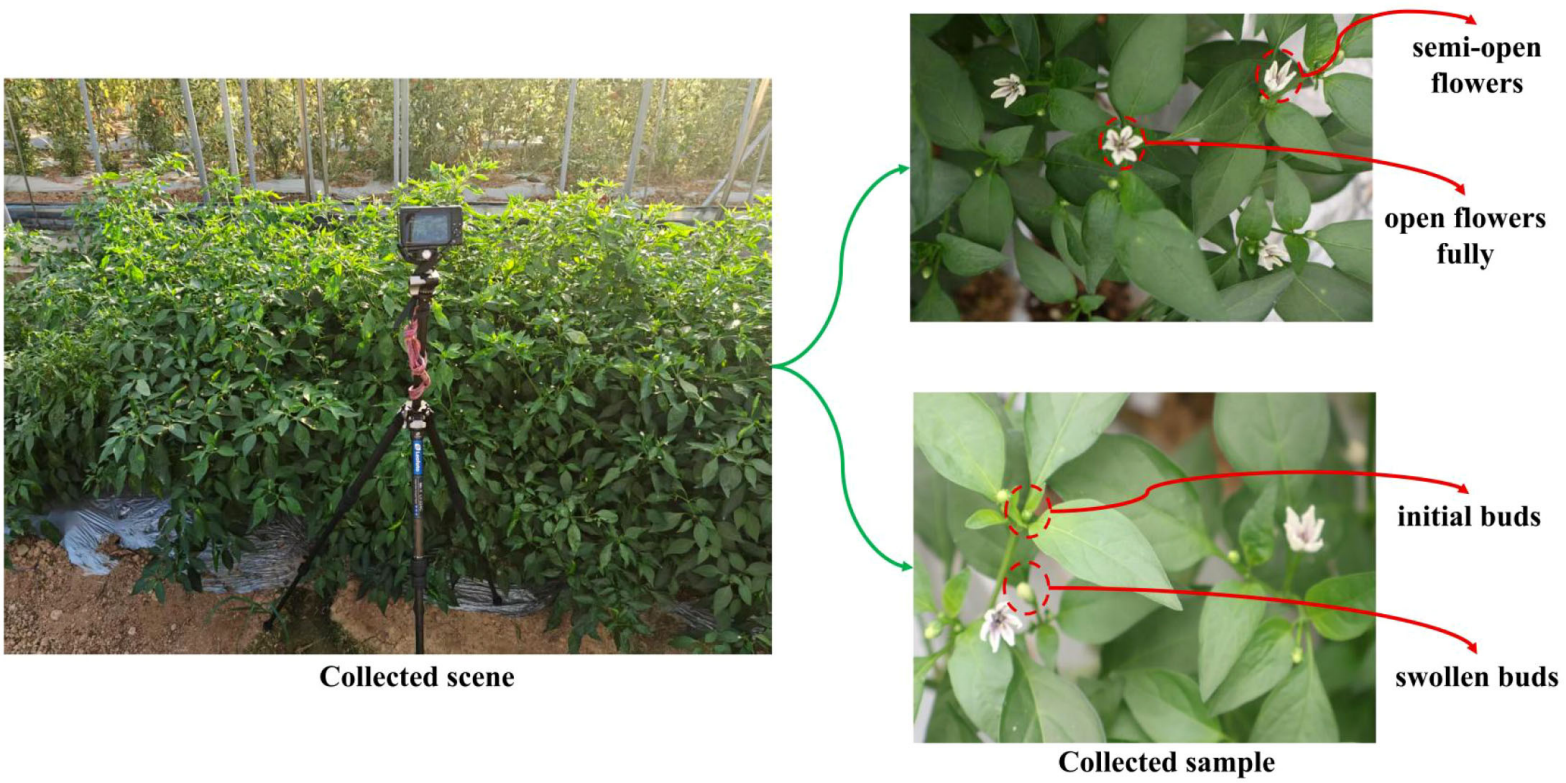
Schematic diagram of image collection for chili pepper flowering period under field natural environment, including the scene of image collection using camera equipment and examples of collected samples.

The image acquisition for this study was conducted at the Vegetable Research Base of Hunan Agricultural University (Changsha, China; 28°11’N, 113°04’E). To construct a high-quality image dataset for the fine-grained identification and developmental stage classification of chili flowers and buds, the “8214upup” mutant, characterized by its typical upward growth habit, was selected as the experimental material. Images were captured using a Canon R50 high-resolution digital camera between June and August 2025, covering the critical phenological stages of the peak flowering period (as shown in **Figure 1**).

Images were acquired using a Canon R5 high-resolution digital camera from June to August 2025, covering the key phenological stages during the peak flowering period (as shown in **Figure 1**). To meet the requirements of agronomic detection and mechanical pollination, the data acquisition time slots were set as 06:00–11:00 and 15:00–17:00 daily. A multi-angle top-down shooting strategy was adopted during the acquisition process: by flexibly adjusting the camera height, focal length, and shooting angles, the floral structures at different developmental stages were systematically recorded, including initial flower buds, swollen flower buds, semi-open flowers, and fully open flowers. This method significantly improved the representativeness and generalization capability of the dataset in terms of morphological diversity, scale variation, and background complexity.

To further characterize the morphological diversity, we analyzed samples covering different phenological stages from initial green flower buds to fully open white flowers. As illustrated in **Figure 1**, the initial flower bud stage is characterized by small green buds with a diameter of 1–3 mm, which are closely attached to the leaf axils and tightly enclosed by sepals. They exhibit high similarity to leaves in both color and scale, making them difficult to identify. In the swollen flower bud stage, the buds obviously expand to 4–8 mm with a plump outline; the petal tips slightly emerge, the pedicels elongate, and the internal anthers begin to mature, making this stage a critical preparation window for mechanical pollination. During the semi-open flower stage, the corolla partially unfolds into a star shape, with white or pale purple petals revealed. The stamens are exposed and release pollen, while the stigma becomes pollination-competent. This stage features a clear morphology and high contrast against the background, which is conducive to image recognition. In the fully open flower stage, the corolla is completely unfolded (with a diameter of 10–15 mm), the stamens and stigma are fully exposed, and the ovary starts to swell. This stage enters the optimal period for pollination and fruit set, with a stable structure and distinct features, making it the most suitable for automated detection and agronomic operations.

**Figure 2:**
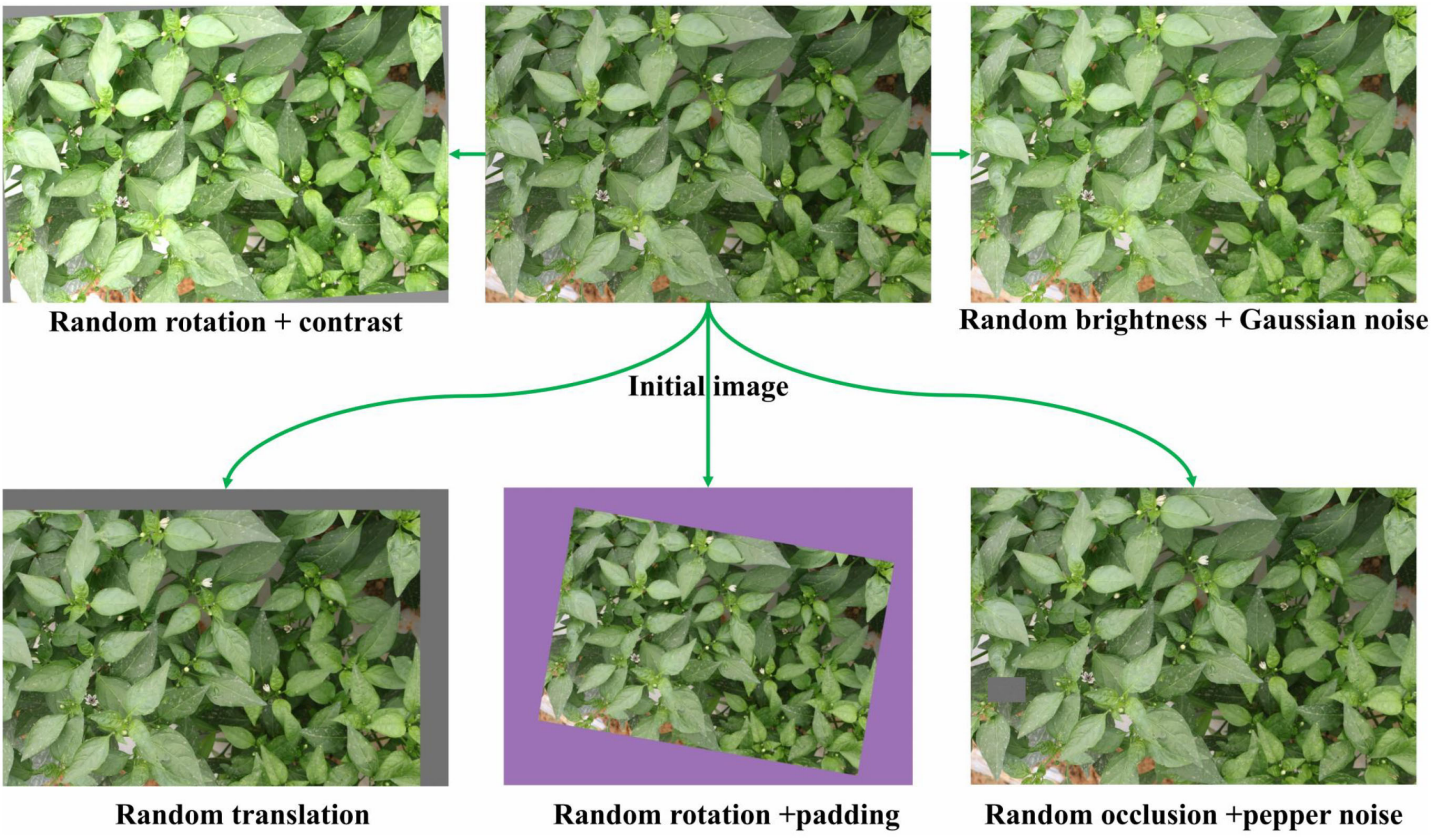
Dataset augmentation processing for chili pepper flowering period, including random rotation, contrast adjustment, brightness adjustment, Gaussian noise injection, translation, and occlusion simulation.

To further bolster the model’s generalization ability and robustness in unstructured field environments, a rigorous data cleaning and augmentation pipeline was implemented. Initially, low-quality samples exhibiting severe motion blur or extreme occlusion were manually filtered out, establishing a benchmark dataset of 1,875 high-resolution original images. Subsequently, a hybrid data augmentation strategy was introduced. Techniques such as random padding with rotation, random brightness adjustment with Gaussian noise, random occlusion with salt-and-pepper noise, geometric translation, and random rotation with contrast adjustment were combined to expand the dataset to 7,274 images (as shown in **Figure 2**). This process aimed to enrich the feature space of the samples, specifically enhancing the model’s adaptability to targets of varying scales. All samples were meticulously annotated using the X-AnyLabeling software to explicitly define the spatial coordinates and posture categories of the flowers. Finally, the dataset was randomly partitioned into a training set (5,819 images), a validation set (727 images), and a test set (728 images) in an 8:1:1 ratio.

To ensure the transparency and reproducibility of the data processing, the detailed stochastic parameter ranges and the specific sample counts for each technique are summarized in **Table 1**. Each augmentation was implemented using Python-based scripts (e.g., OpenCV and Albumentations) with randomized parameters (e.g., random rotation angles $\theta \in [-15^{\circ },+15^{\circ }]$ and stochastic brightness scales *α* ∈ [0.8,1.2]). This stochasticity ensures that each augmented sample represents a unique feature distribution, thereby enhancing the model’s resistance to overfitting even when variants of the same source image exist across different subsets.

**Table 1:** Detailed parameters and sample distribution of randomized data augmentation methods.

Augmentation method	Parameter range (Stochastic)	Final count
Initial Images	–	1,875
Random rotation + contrast	$\theta \in [-15^{\circ },+15^{\circ }]$, Contrast ∈ [0.8,1.2]	1,080
Random brightness + Gaussian noise	Brightness ∈ [0.8,1.2], *σ* ∈ [0,10]	1,080
Random translation	Translation ratio ∈ [0.05,0.1]	1,080
Random rotation + padding	$\theta \in [-15^{\circ },+15^{\circ }]$, Constant fill	1,080
Random occlusion + pepper noise	Size [20 × 20,50 × 50], Prob = 0.05	1,079
Total	–	7,274

### 2.2 Overall roadmap

**Figure 3:**
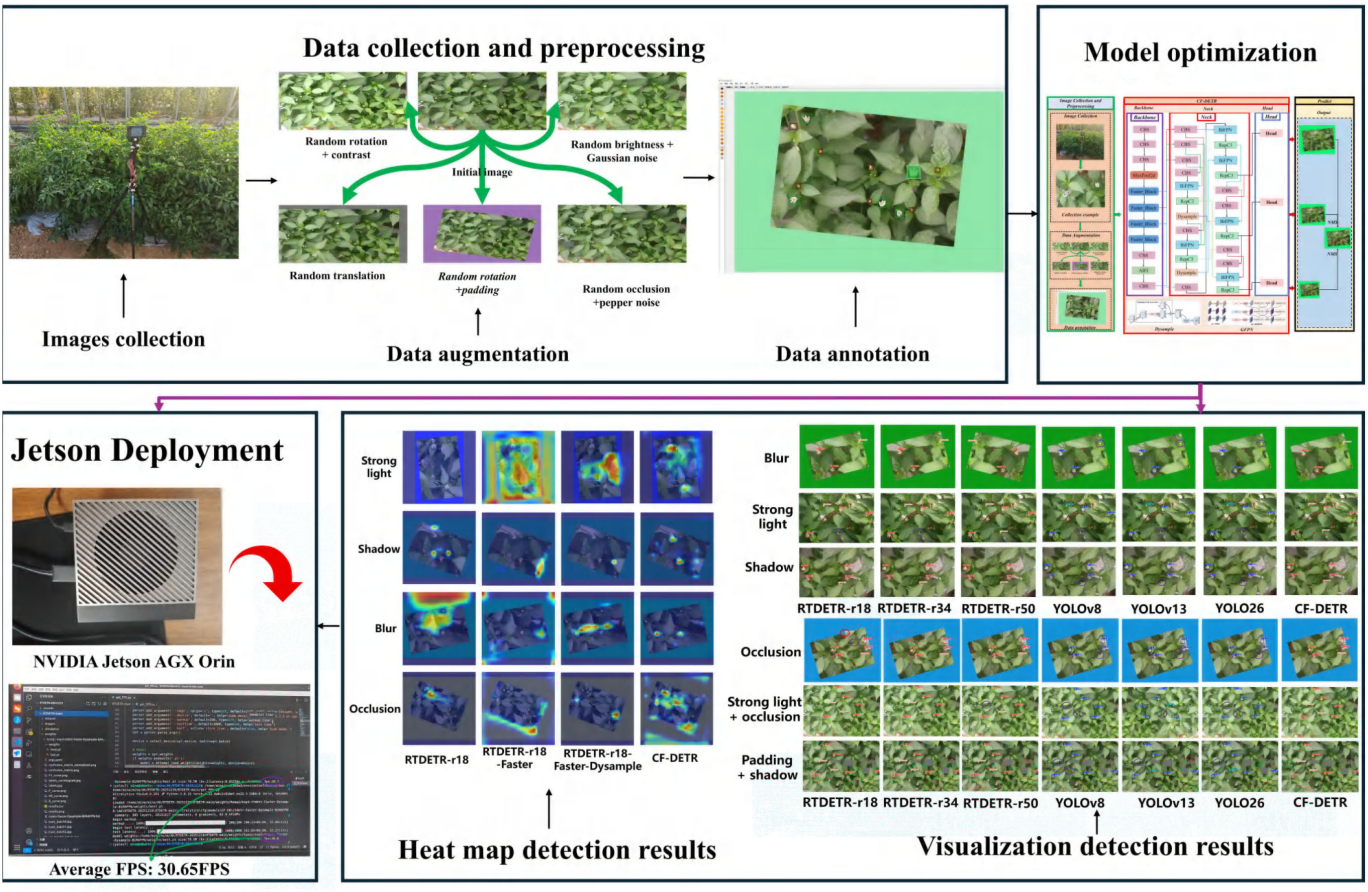
Overall technological route of the proposed method, covering the complete research flow from data acquisition and preprocessing, optimal design of the CF-DETR model, to final deployment on the Jetson edge platform and visual analysis.

**Figure 3** illustrates the comprehensive technical roadmap for chili flowering period detection and pose estimation proposed in this study. This framework aims to leverage end-to-end deep learning technologies to overcome the challenges of precise perception and real-time processing of minute targets in unstructured and complex agricultural environments. The research workflow systematically encompasses key stages comprising scene image acquisition and preprocessing, lightweight network architecture optimization, model training with ablation validation, and embedded terminal deployment.

First, addressing environmental characteristics such as dynamic lighting variations and high-density foliage occlusion in the field, multi-view and multi-temporal chili flower images were collected and meticulously annotated to construct a high-quality benchmark dataset comprising training, validation, and testing sets. Data augmentation strategies, including random rotation, brightness adjustment, and Gaussian noise injection, were introduced to further enhance sample diversity and the model’s generalization potential.

Second, an improved CF-DETR model was proposed. Using RT-DETR-r18 as the baseline architecture, this model innovatively incorporates FasterNet to replace the original backbone network. Simultaneously, the DySample dynamic upsampling operator is adopted to reconstruct the neck network. A BiMAFPN (Bidirectional Multi-scale Attention Feature Pyramid Network) feature fusion structure was designed and integrated. Systematic reconstruction and innovation were carried out across three dimensions, namely backbone feature extraction, feature fusion mechanisms, and upsampling strategies, aiming to resolve core pain points including high missed detection rates for minute targets and insufficient feature extraction.

Finally, after iterative training and parameter optimization, the model’s feature focusing capability in complex backgrounds was visually verified using Grad-CAM heatmaps, and detection precision was evaluated through comparative experiments. The optimized model was then deployed onto the NVIDIA Jetson AGX Orin embedded edge computing platform via the TensorRT acceleration engine. Tests indicate that the system achieves a real-time detection speed of approximately 32.6 ms per frame (corresponding to 30.65 FPS), successfully realizing high-precision, low-latency visual perception on resource-constrained edge devices. This provides reliable visual guidance and decision support for intelligent flowering monitoring and pollination robots.

### 2.3 Chili flowering period classification and detection model

In the current field of object detection, RT-DETR (Real-Time Detection Transformer), with its Transformer-based global attention mechanism and end-to-end detection capabilities, has demonstrated superior performance in processing conventional targets, effectively overcoming the limitations of traditional Convolutional Neural Networks (CNNs) in modeling long-range dependencies. However, the chili flowering period detection task in unstructured greenhouse environments presents highly specific challenges. Specifically, targets are minute (Tiny Objects), growth is extremely dense (Dense Distribution), and interference from foliage occlusion and dynamic lighting is severe.

Addressing the biological characteristics of chili flowers, particularly their fragmented petals and weak bud textures, as well as the computational redundancy and insufficient spatial feature extraction efficiency in the original RT-DETR-r18 backbone, this study first introduces the lightweight FasterNet to replace the original backbone. Leveraging its innovative Partial Convolution (PConv) mechanism, which performs convolution on only a subset of input channels, FasterNet significantly reduces computational load and memory access costs while optimizing spatial feature extraction efficiency. This enables the acute capture of edge texture information for minute flowers and buds, laying a solid foundation for subsequent processing.

Building on this, to mitigate the critical impact of detail blurring and noise introduced by traditional interpolation upsampling on small target detection, the DySample operator is adopted to reconstruct the neck network. By utilizing a point-sampling mechanism based on learned offsets to replace kernel-based dynamic convolution, this approach achieves adaptive resampling and high-fidelity transmission of feature maps. This significantly improves reconstruction quality and localization precision for minute targets such as nascent buds.

Furthermore, facing the challenge of scale variation caused by drastic differences in chili flower growth stages, a BiMAFPN feature fusion structure was designed and introduced. This structure integrates multi-scale attention mechanisms into the bidirectional cross-scale connection foundation of BiFPN. By strengthening the interactive fusion and adaptive weighting of deep semantic information and shallow high-resolution details, it effectively resolves feature loss issues and reduces missed detection rates.

**Figure 4:**
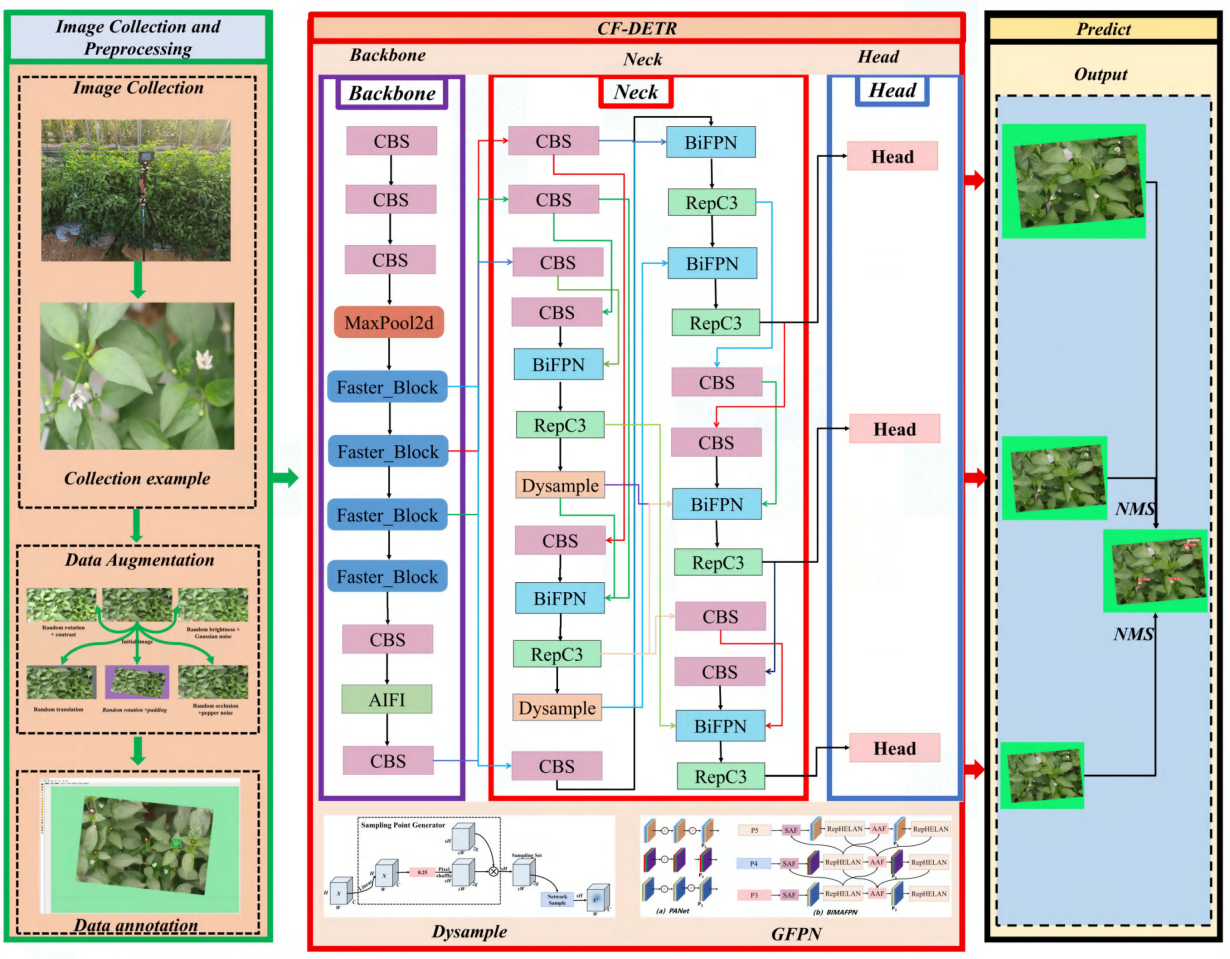
Network architecture of the improved CF-DETR model, including the Backbone, Neck (integrating BiFPN and Dysample), and detection Head.

Finally, these improvements, combined with the inherent NMS-free (Non-Maximum Suppression-free) decoding characteristic of RT-DETR, fundamentally avoid false positives and missed detections caused by candidate box suppression strategies in dense overlapping scenarios, realizing precise counting and localization of high-density chili flower clusters. The structure of the improved CF-DETR model is shown in **Figure 4**. Through the synergistic effect of the three core improvements described above, this model achieves high-precision and high-efficiency perception of chili flower postures in complex unstructured environments.

#### 2.3.1 Fasternet backbone network

In the task of chili flowering period classification and recognition, the biological characteristics of chili flowers, including their fragmented petals, weak bud textures, and high similarity to the background, impose stringent requirements on the network’s spatial feature capture capabilities. The original RT-DETR-18 backbone consists of a series of standard convolutional layers. During image processing, standard convolution performs indiscriminate operations on all input channels. However, in deep feature maps, adjacent channels often contain highly similar redundant information. This excessive redundancy not only fails to improve detection accuracy but may also lead to the “dilution” or submersion of minute floral edge texture features amid substantial repetitive background noise, thereby limiting the model’s representation capability for tiny targets.

**Figure 5:**
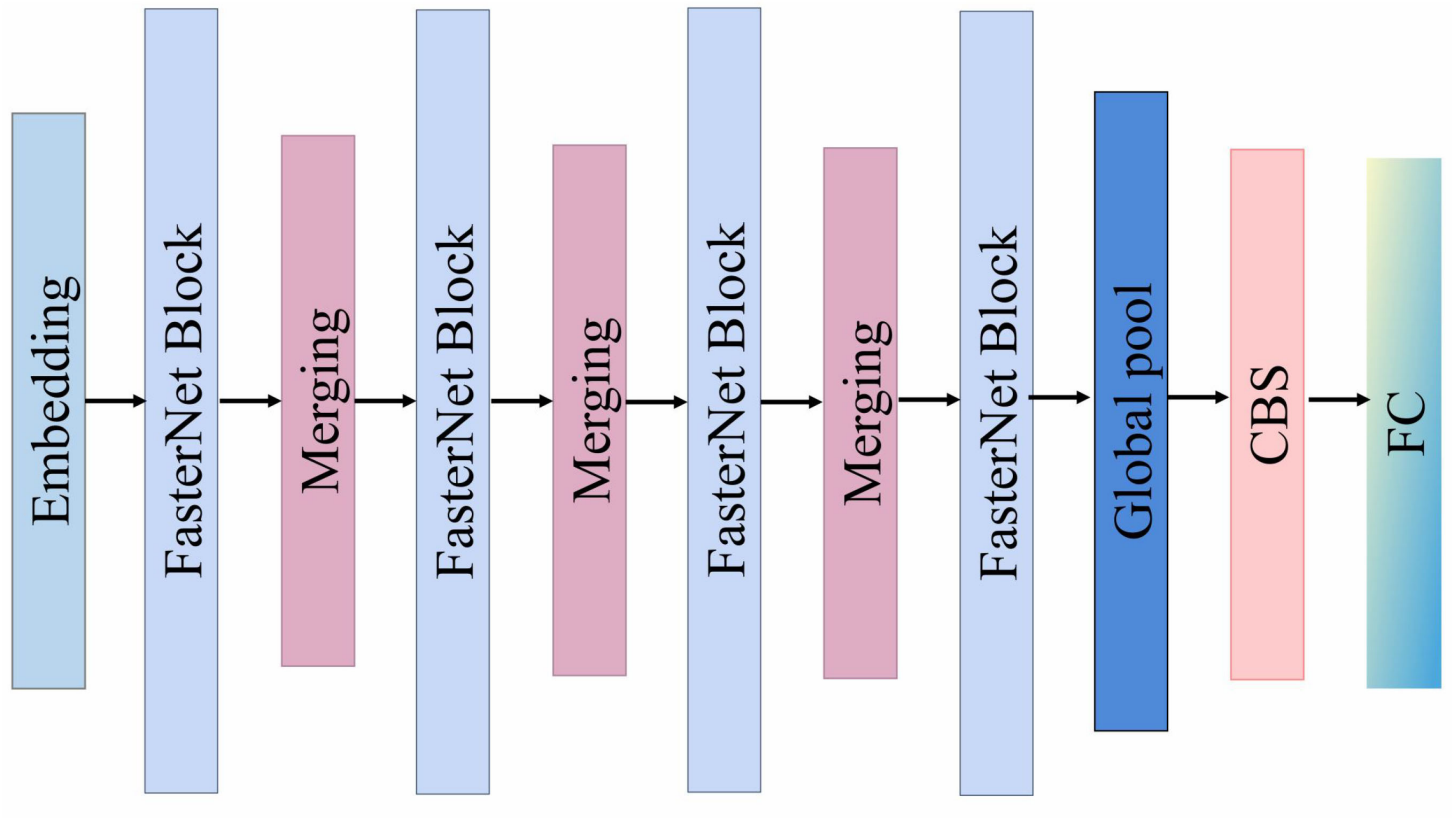
Structure of the FasterNet backbone network for feature extraction, illustrating the connections of internal modules including Embedding, Merging, and FasterNet Block.

To address this, this study introduces the FasterNet backbone (as shown in **Figure 5**) to replace the original backbone network, reconstructing the feature extraction process based on its core Partial Convolution (PConv) mechanism. The fundamental concept of PConv leverages the similarity between feature map channels by abandoning the traditional mode of processing all channels simultaneously. Instead, it performs convolution operations on only a representative subset of channels to extract spatial features, while the remaining channels are kept unchanged to maintain the original information flow.

Based on convolutional arithmetic theory, Floating Point Operations (FLOPs) are used to quantify the complexity and redundancy of the feature extraction process. The computational complexity comparison between Standard Convolution (SC) and Partial Convolution (PConv) can be expressed by Equations 1, 2, respectively:

(1) $$F_{SC}=h\times w\times c\times c\times k^{2}$$

(2) $$F_{PC}=h\times w\times c_{p}^{2}\times k^{2}$$

here *h* and *w* represent the height and width of the feature map; *c* denotes the number of input channels; *k* is the kernel size; and cp is the number of partial channels involved in the convolution operation. In this study, we set *c_p_/c* = 1*/*4, meaning only 1/4 of the channels are used for feature extraction. Consequently, the FLOPs of PConv are reduced to only 1/16 of those of standard convolution. Based on Equations 1, it can be deduced that as layers stack, the vector space dimensionality grows exponentially. The vector space dimension is (*d/*2)2*l*, and the model’s star operation count is 5. When the input channel count is 128, the implicit feature dimension can reach approximately 9032 (derivation available in [26]). This immense high-dimensional spatial property enables FasterNet to project high-frequency detailed features, such as chili petal edges and stamen orientations, into a high-dimensional space for decoupling, thereby effectively resolving feature ambiguity caused by minute target sizes and significantly improving pose classification sensitivity. Furthermore, calculating the Memory Access (MA) of PConv as shown in Equation 3:

(3) $$G_{PC}=h\times w\times 2c_{p}+k^{2}\times c_{p}^{2}$$

Similarly, when *c_p_*= 1*/*4, the memory access of PConv is also only 1/16 of that of standard convolution. Theoretical calculations from (Equations 1–3) demonstrate that introducing PConv drastically reduces redundant operations at the feature extraction level. For chili flower detection, this numerical reduction implies more than just computational compression; it fundamentally represents a “purification” of the feature extraction strategy. By convolving only 1/4 of the critical channels, the network avoids dispersing attention across massive repetitive redundant channels, thereby focusing the receptive field more intensively on parts containing rich spatial details. This mechanism effectively optimizes spatial feature extraction efficiency, enabling the network to more acutely capture the edge texture information of minute flowers and buds. It resolves the issue of insufficient spatial information extraction caused by feature redundancy in the original network, laying a solid and purer feature foundation for subsequent classification and localization.

#### 2.3.2 DySample dynamic upsampling module

In the chili flowering period classification task, the original YOLOv8n network employs Nearest Neighbor Interpolation for upsampling in the neck feature fusion stage to adjust feature map dimensions. However, given the biological characteristics of fine chili petals and weak textures of nascent buds, nearest neighbor interpolation exhibits significant drawbacks: it merely replicates the nearest pixel values to enlarge the image, failing to supplement context information from surrounding pixels. This often leads to the loss of high-frequency details (e.g., petal edges, stamen textures) and introduces aliasing or blurring effects, making minute bud features difficult to preserve effectively.

**Figure 6:**
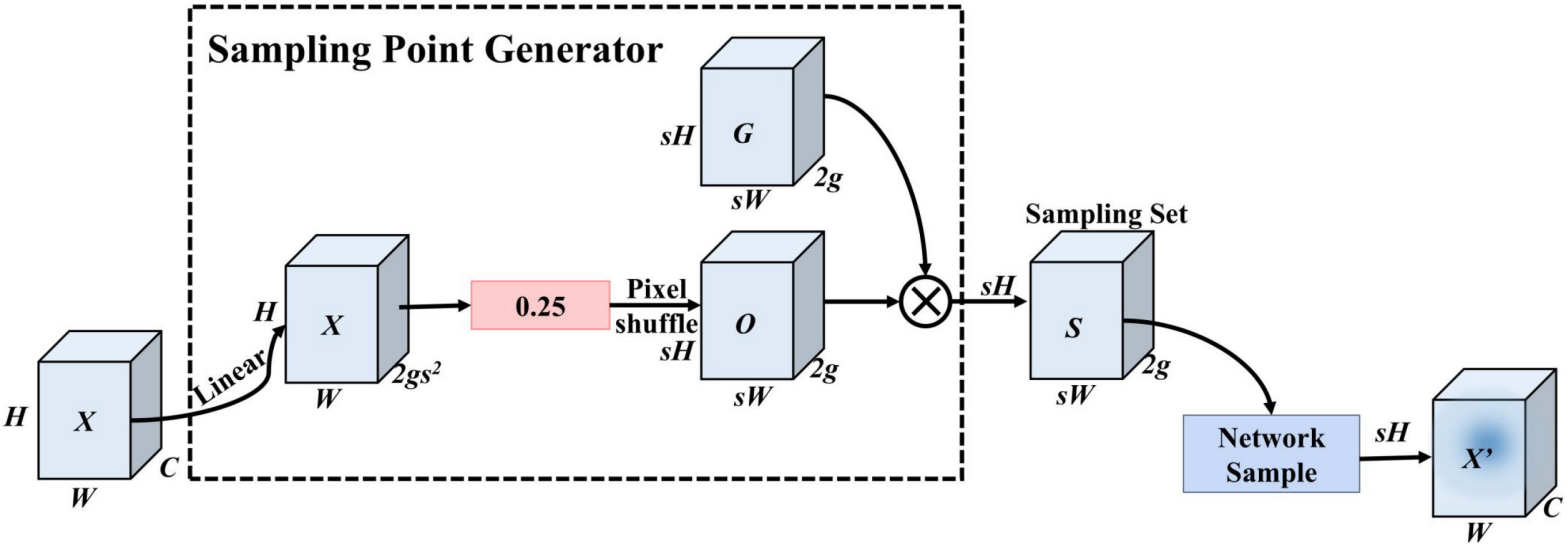
Structure of the Dysample upsampling module, illustrating the internal mechanism including sampling point generation and pixel reorganization.

Therefore, this study introduces the DySample (Dynamic Sampling) operator (shown in **Figure 6**) into the neck network to replace traditional nearest neighbor interpolation. DySample is an ultralightweight dynamic upsampling method that bypasses the computationally expensive kernel-based dynamic convolution in favor of a Point Sampling mechanism. Its core advantage lies in its ability to adaptively learn sampling point position offsets based on feature information from different regions of the chili flower sample image (e.g., flower edges vs. background leaves), thereby achieving high-fidelity recovery of features.

The principle of this upsampling operator is as follows: Given an input feature map X of size C×H×W, where C, H, and W represent the number of channels, height, and width, respectively. The module reconstructs features through the following steps: First, to generate sampling offsets at a low computational cost, a linear layer is used to process the input features. To match the upsampling scale factor s, the output channels are set to 2s2. To prevent local overlap of s2 sampling positions, a Static Scope Factor (coefficient 0.25) is introduced to initially constrain the offsets. Then, via Pixel Shuffle, the output is reshaped to 2×sH×sW, resulting in the offset O relative to the original sampling grid G. The initial offset generation and sampling position calculation are formulated as in Equations 4, 5.

(4) O=0.25 Linear(X)

(5) S=G+O

Here, Linear(x) denotes the linear transformation of input feature x to generate base offsets; 0.25 is the static scope factor used to constrain offset magnitude to avoid sampling position overlap; *G* is the original sampling grid; *O* is the offset; and *S* is the final set of sampling positions. Subsequently, using PyTorch’s grid sample function, the input feature *X* is resampled to the target feature map *X*^′^ based on the calculated positions *S* in Equation 6.

(6) $$X^{\prime }=\text{gridsample}(X,S)$$

However, relying solely on static constraints may still lead to sampling point overlap when processing complex textured backgrounds like chili flowers, resulting in detection errors. To further enhance feature capture for minute targets (e.g., buds), DySample introduces a Dynamic Scope Factor. This mechanism generates dynamic adjustment coefficients by linearly projecting input features. One side is constrained by a static scope of 0.5, while the other remains unconstrained, allowing flexible adjustment of the offset O to reduce overlap. The improved offset calculation formula is Equation 7:

(7) $$O=0.5\text{ Sigmoid}({\text{Linear}}_{1}(X))\cdot {\text{Linear}}_{2}(X)$$

By introducing the DySample module, the network no longer mechanically enlarges feature maps but realizes adaptive resampling through the learning mechanism of Equations 4–7. For weakly textured chili buds, DySample can more precisely focus feature points on target regions rather than background noise, significantly improving detail reconstruction quality during upsampling. This improvement overcomes the detail blurring caused by traditional interpolation, providing higher-quality spatial feature information for the subsequent RT-DETR decoding head, thereby fundamentally enhancing localization precision and detection performance for high-density, minute chili flower clusters.

### 2.4 BiMAFPN feature extraction module

**Figure 7:**
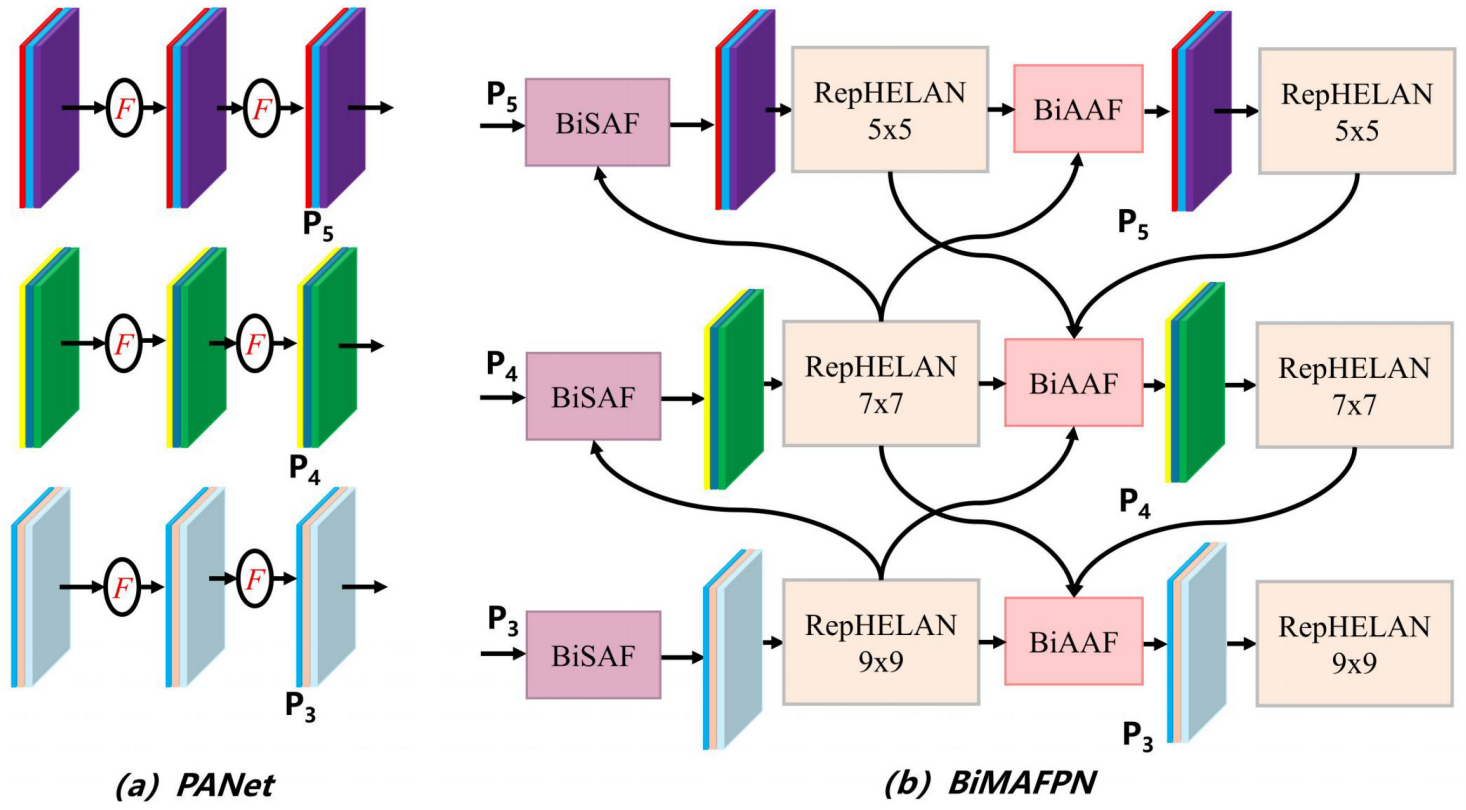
Structural diagram of the BiMAFPN feature fusion module, comparing the feature pyramid fusion strategies between **(a)** traditional PANet and **(b)** the proposed BiMAFPN.

In unstructured field environments, the task of chili flowering period classification and recognition faces severe scale variation challenges. Influenced by shooting distance, growth angle, and foliage occlusion, chili flowers exhibit significant multi-scale characteristics in images. Moreover, as typical minute targets, their key classification features (e.g., stamen orientation, petal edges, flowering degree) are highly prone to information loss during multiple downsampling processes in deep networks. Traditional single-path feature extraction struggles to balance deep semantic information with shallow geometric details across multiple scales. To this end, this study proposes a Bidirectional Multi-scale Attention Feature Pyramid Network (BiMAFPN), as shown in **Figure 7**. This architecture innovatively integrates the Multi-branch Auxiliary Feature Pyramid Network (MAFPN) with the Bidirectional Feature Pyramid Network (BiFPN). While retaining BiFPN’s efficient cross-scale weighted fusion capability, it introduces an auxiliary backbone branch to inject rich shallow high-resolution gradient information into the neck network. Specifically, the auxiliary branch preserves fine-grained geometric features of small flowers (such as petal contours and stamen textures) that are easily lost in deep downsampling, while the bidirectional attention fusion mechanism reweights multi-scale features to suppress irrelevant background clutter (e.g., soil, leaves, and light shadows) and focus on target-related details. This design effectively enhances the model’s perception of minute flower targets and suppressing background noise.

The core advantage of BiMAFPN lies in its weighted cross-scale connection mechanism, which breaks the limitation of equal-weight fusion in traditional FPNs. By introducing learnable weight parameters, the network can adaptively focus on feature levels that contribute more to flowering period classification. For feature fusion at layer *l*, defining Plin as the input feature and Plout as the output feature, the fusion process can be represented by the modified weighted normalized fusion Equation 8.

(8) $$O_{l}=W_{\left(l,1\right)}^{T}O_{i-l}\;W_{\left(l,2\right)}^{T}O_{i-l}(O_{i-l}\in {\mathbb{R}}^{{\left(\frac{d}{\sqrt{2}}\right)}^{2l}})$$

Where $O_{i}{-}_{l}$ is the input feature of layer $l-1;W_{\left(l,1\right)}^{T}$, represent learnable fusion weights; represents the scale transformation factor of feature dimensions. In practical applications, to achieve fast computation, a fast normalized fusion strategy is typically adopted. For intermediate fusion nodes, the output feature Pout is calculated as shown in Equation 9.

(9) $$P^{mt}=\text{Conv}(\frac{w_{1}\cdot P^{in}+w_{2}\cdot Resiec(P^{ut})+w_{3}\cdot Resiec(P^{md})}{w_{1}+w_{2}+w_{3}+\in })$$

Here, *P^in^*,*P^ut^*,*P^md^* represent input features from different levels; *w*_1_*,w*_2_*,w*_3_ are non-negative learnable fusion weights, ensuring the network can automatically assign attention based on the importance of features at different levels; *∈* is a small constant for numerical stability (usually 0.0001); and the Resize operation unifies resolution via upsampling or downsampling. Through this weighting mechanism, the model can automatically balance deep semantic information (e.g., category features of buds) and shallow geometric information (e.g., edge textures of petals), thereby precisely capturing the subtle differences in chili flowers.

To further strengthen the preservation of shallow details, we integrated a BiSAF (Bidirectional Shallow Attention Fusion) module into BiMAFPN. This module aims to directly inject shallow high-resolution features, which retain rich localization details from the backbone network, into the fusion path. Assuming *Pn* − 1*,Pn* are backbone feature layers of different resolutions, and $Pn+1^{\prime }$ is the deep feedback feature, the output feature calculation logic of BiSAF is as shown in Equation 10.

(10) $$P_{out}=\text{Conv}(\text{Attan}(P_{shallow})+\text{UpSample}(P_{deep}))$$

### 2.5 Test environment and evaluation metrics

#### 2.5.1 Test environment

To ensure the rigor, reproducibility, and objectivity of the comparative analysis, a unified and standardized computing platform was constructed to conduct all model training, ablation studies, and inference tests. The hardware infrastructure was equipped with an Intel^®^ Core™ i7-13700KF processor and an NVIDIA GeForce RTX 3090 Ti graphics card (24 GB VRAM) to provide sufficient computational power for complex deep learning tasks. The software environment was built on the Windows 10 Professional (64-bit) operating system, with algorithms implemented using the Python programming language and the PyTorch deep learning framework, deployed within the PyCharm Community Edition integrated development environment. To maintain strict experimental control, all comparative experiments utilized identical dataset partitioning schemes. Unless specified as a variable under investigation, all hyperparameters and default configurations were kept consistent throughout the process. The detailed training parameter settings are presented in **Table 2**.

**Table 2:** Training parameters.

Training parameter	Value or type
Input image size	640 × 640 × 3
Optimizer	SGD
Optimizer momentum	0.937
Python version	3.9.20
Initial learning rate	0.01
Patience	100
Batch size	4
Epoch	200

#### 2.5.2 Model evaluation metrics

Given the challenges inherent to chili flowering detection, specifically the minute target size and susceptibility to complex background interference such as foliage occlusion and dynamic lighting, this study selected Precision (P), Recall (R), and mean Average Precision (mAP) as the core metrics to comprehensively and objectively quantify the model’s performance in pose recognition and localization tasks.

Specifically, Precision characterizes the accuracy of positive sample predictions, reflecting the algorithm’s capability to suppress false positives. Recall measures the model’s ability to retrieve all ground-truth targets from complex backgrounds, indicating its robustness against missed detections. Furthermore, mAP50 (the mean AP at an Intersection over Union (IoU) threshold of 0.5) and mAP 50-95 (the mean AP averaged over IoU thresholds from 0.5 to 0.95) were introduced to evaluate the trade-off between localization precision and classification accuracy. The mathematical definitions for these metrics are in Equations 12–14. Floating Point Operations (FLOPs) quantifies the total number of arithmetic operations performed by a model during inference, measured in Giga (10^9^) operations. It directly reflects the computational complexity and hardware resource consumption of the model, where lower FLOPs indicate higher computational efficiency and suitability for deployment on edge devices with limited computing power. Parameters (Params) represent the total number of trainable weight parameters in the model, which determines the model size and memory usage. Frames Per Second (FPS) measures the number of image frames a model can process per second, with the unit of frames per second (f/s). It directly evaluates the real-time performance of the model in practical deployment scenarios. Higher FPS means the model can complete target detection faster, meeting the real-time requirements of automated agricultural operations such as mechanical pollination and harvesting.

(11) $$P=\frac{C_{TP}}{C_{TP}+C_{FP}}\times 100\%$$

(12) $$R=\frac{C_{TP}}{C_{TP}+C_{FN}}\times 100\%$$

(13) $$AP=\int_{0}^{1}P(R)\text{ d}R$$

(14) $$mAP=\frac{1}{n}\sum_{i=1}^{n}AP_{i}$$

Where *C*_TP_ (True Positives) denotes the number of chili flower samples correctly identified and localized by the model (i.e., IoU between the predicted box and ground truth exceeds the threshold); *C*_FP_ (False Positives) represents the number of false detections, such as background noise (e.g., leaf reflections) or misclassified categories; and *C*_FN_ (False Negatives) indicates the number of ground-truth chili flower targets missed by the model. AP (Average Precision) represents the area under the Precision-Recall (P-R) curve, serving as a comprehensive metric for single-class detection performance.

## 3 Results and discussion

To validate the effectiveness of the proposed CF-DETR model (Note: Changed from DCFP-YOLO to CF-DETR to match your previous abstract, please confirm the model name) in chili flower pose recognition, a comprehensive evaluation was conducted from four dimensions: ablation studies, comparative experiments, visualization of detection results, and heatmap analysis. This multi-faceted approach provided a holistic assessment of the model’s performance in terms of detection accuracy, computational efficiency, and deployment capability.

### 3.1 Ablation experiment

Ablation experiments serve as a critical validation method in deep learning model optimization, systematically removing or adjusting model components to assess their individual contributions to overall performance. To quantify the specific impact of each improvement module and their synergistic effects, this study utilized RTDETR-r18 as the baseline model. A stepwise integration strategy was employed to conduct ablation experiments involving the FasterNet backbone (Module A), the DySample dynamic upsampling operator (Module B), and the BiMAFPN feature fusion network (Module C). The experimental results are summarized in **Table 3**.

**Table 3:** CF-DETR ablation experiment results.

Models	Modules			Evaluation metrics						
	A	B	C	*P* (%)	*R* (%)	mAP_50_ (%)	mAP_50-95_(%)	FLOPs(G)	Params(M)	FPS(f/s)
RTDETR-r18	–	–	–	93.2	78.5	81.5	62.4	56.9	19.8	80.0
RTDETR-r18	✓	–	–	94.4	77.9	82.9	63.8	49.5	16.7	68.0
RTDETR-r18	–	✓	–	93.9	77.6	81.9	61.1	57.0	19.8	67.2
RTDETR-r18	–	–	✓	95.4	77.1	81.9	63.2	57.5	20.1	62.8
RTDETR-r18	✓	✓	–	**94.5**	78.0	82.7	64.0	49.5	16.8	64.1
RTDETR-r18	✓	–	✓	93.2	77.8	81.3	60.2	62.0	20.1	58.6
RTDETR-r18	✓	✓	✓	94.1	**78.7**	**83.5**	**64.5**	62.0	20.1	56.3

Note: ✓ indicates the module is used; “–” indicates not used. All metrics are measured on a single NVIDIA RTX 3090 GPU.

Bold font means the best value.

As demonstrated by the ablation experiments in **Table 3**, initially, the independent integration of Module A, the FasterNet-based backbone, triggered a fundamental restructuring of detection accuracy alongside a significant reduction in computational complexity. This module successfully curtailed floating-point operations to 49.5 G and compressed the parameter count to 16.7 M, while concurrently elevating precision to 94.4 percent. However, this high precision achieved through lightweight feature extraction came at the expense of a marginal decline in recall, suggesting the suppression of feature responses for certain ambiguous targets. When Module B, representing DySample dynamic upsampling, or Module C, representing BiMAFPN feature fusion, were introduced independently, the model experienced fluctuations in precision and the stringent mAP50–95 metric, occasionally accompanied by a rebound in computational overhead. This phenomenon, caused by the absence of matching high-quality front-end feature inputs, underscores the limitations of isolated structural modifications in complex agricultural scenarios.

Further exploration of the coupling gains among combined modules uncovers a pronounced positive synergy between Module A and Module B. Building upon the highly efficient local features provided by Module A, Module B leverages a point-sampling-based dynamic offset learning mechanism to effectively preserve the edge topological information of minute targets. This specific combination drove precision to a global peak of 94.5 percent and advanced mAP50–95 to 64.0 percent, all while strictly maintaining floating-point operations at the ultra-low level of 49.5 G. This successfully realizes a profound integration of architectural lightweighting and high-precision localization. In stark contrast, the combination of Module A and Module C, despite attempting to enrich multi-scale context via the feature pyramid, suffered from insufficient feature alignment due to the lack of upsampling fidelity. This architectural mismatch precipitated a significant degradation in mAP50-95, exposing the negative interference between incompatible structural paradigms.

Ultimately, when Module C with its bidirectional cross-scale attention mechanism was seamlessly integrated into the collaborative framework of A and B, the model attained an optimal Pareto balance between perceptual accuracy and computational efficiency. The complete architecture forcefully compensated for the recall deficiencies induced by early-stage lightweighting, propelling it to 78.7 percent, while concurrently securing peak values for mAP50 and mAP50–95 at 83.5 percent and 64.5 percent, respectively. Although this globally optimal structure registers a modest resurgence in floating-point operations and parameters to 62.0 G and 20.1 M, it not only achieves a substantive leap across all accuracy metrics relative to the baseline but also sustains a robust high-speed inference capability of 56.3 frames per second. This progressive empirical analysis profoundly demonstrates that superior detection performance stems not from the indiscriminate stacking of components, but from the deep mechanistic coupling among highly efficient feature extraction (A), high-fidelity spatial transmission (B), and adaptive semantic fusion (C), thereby providing a solid algorithmic foundation for deployment on resource-constrained terminals.

### 3.2 Comparative experiments

#### 3.2.1 Comparison test of different backbone networks replacement

To systematically investigate the impact of backbone network substitution on the performance of the pepper flowering stage classification model, this study designed three sets of comparative experiments. Specifically, the original YOLOv11n backbone was replaced by MobileNetV4, StarNet, and FasterNet, respectively, and their performances were benchmarked against the baseline model. The experimental results are presented in **Table 4**.

**Table 4:** Comparison of model performance for adding different backbone networks.

The replaced backbone	*P* (%)	*R* (%)	mAP_50_ (%)	mAP_50−95_ (%)
Initial backbone	93.2	**78.5**	81.5	62.4
MobileNetV4	94.9	77.7	82.2	61.1
StarNet	**95.1**	76.6	81.7	60.9
FasterNet	94.4	77.9	**82.9**	**63.8**

Bold font means the best value.

In terms of Precision, all three backbone substitution schemes demonstrated superior feature discrimination capabilities compared to the original network. StarNet achieved the peak Precision of 95.1%, while MobileNetV4 and FasterNet reached 94.9% and 94.4%, respectively, all surpassing the 93.2% of the original backbone. However, regarding Recall, all improved lightweight backbones exhibited varying degrees of decline compared to the baseline’s 78.5%. This suggests that lightweight architectures possess certain inherent limitations in preserving the integrity of deep semantic features.

Nevertheless, in key metrics evaluating comprehensive model performance, FasterNet demonstrated a significant advantage. Its mAP50 reached 82.9%, which is not only higher than MobileNetV4 (82.2%) and StarNet (81.7%) but also represents an improvement of 1.4 percentage points over the original backbone. Crucially, in the mAP50–95 metric, which reflects high-precision localization capability, FasterNet achieved the best score of 63.8%, outperforming MobileNetV4 (61.1%) and StarNet (60.9%). These results substantiate that the unique Partial Convolution (PConv) mechanism of FasterNet effectively preserves spatial detail features critical for tiny object localization, thereby achieving an optimal balance between detection precision and localization quality. Consequently, FasterNet was selected as the backbone improvement scheme for this study.

#### 3.2.2 Comparative experiments incorporating different upsampling methods

To systematically explore the influence of different upsampling operators on model performance, three sets of comparative experiments were designed based on the FasterNet backbone (YOLOv11n+A). The original upsampling module was replaced by CARAFE and DySample, respectively. The comparative results are shown in **Table 5**.

**Table 5:** Performance comparison of models with different upsampling operators replaced.

Different upsampling methods	*P* (%)	*R* (%)	mAP_50_ (%)	mAP_50−95_ (%)
RTDETR-r18 + A	94.4	77.9	**82.9**	63.8
CARAFE	**95.2**	77.3	81.9	61.4
Dysample	94.5	**78.0**	82.7	**64.0**

Bold font means the best value.

The experimental results indicate that although the CARAFE operator increased the model’s Precision to 95.2%, its Recall dropped to 77.3%. More critically, its mAP50 and mAP50–95 were only 81.9% and 61.4%, respectively, both lower than the baseline model without upsampling improvement (YOLOv11n+A). This implies that the CARAFE operator, when dealing with pepper flower targets with weak textures, may introduce excessive noise due to over-reconstruction of features, thereby impairing localization accuracy. In contrast, the DySample operator demonstrated a more robust and comprehensive performance improvement. Although its Precision (94.5%) was slightly lower than CARAFE, it successfully raised the Recall to 78.0%, effectively mitigating the missed detection problem for tiny targets. Furthermore, DySample achieved 64.0% in mAP50-95, realizing a further performance gain over the baseline’s 63.8%. This demonstrates that DySample’s dynamic offset mechanism based on point sampling can more accurately recover subtle structural information in feature maps, improving bounding box regression quality. Considering the enhancement in high-precision localization and the improvement in Recall, DySample was determined as the optimal upsampling scheme for the application scenario of this study.

#### 3.2.3 Replace different feature fusion methods

To systematically investigate the impact of different feature fusion methods, six sets of comparative experiments were conducted based on the integration of the FasterNet backbone and DySample upsampling. The original feature fusion methods was replaced by combinations involving RFPN, MFMAFPN, BiFPN, MAFPN, and BiMAFPN, respectively. The results are shown in **Table 6**.

**Table 6:** Performance comparison of models with different feature fusion schemes.

Replaced feature fusion methods	*P* (%)	*R* (%)	mAP_50_ (%)	mAP_50−95_ (%)
RTDETR-r18 + A + B	94.5	78.0	82.7	64.0
RFPN	93.6	**79.4**	82.5	64.2
MFMAFPN	94.0	78.0	82.6	63.0
BiFPN	94.5	77.6	81.6	63.4
MAFPN	**94.8**	77.9	82.6	64.0
BiMAFPN	94.1	78.7	**83.5**	**64.5**

Bold font means the best value.

In terms of Precision, MAFPN performed most prominently, reaching 94.8%, while BiFPN also maintained a high level of 94.5%, indicating that simply strengthening the connection paths of the feature pyramid helps improve the model’s discriminative ability for target categories. However, both structures showed relative deficiencies in Recall, with BiFPN dropping to 77.6%, suggesting potential risks of missed detections when handling multi-scale tiny targets. Conversely, although RFPN increased Recall to 79.4%, it came at the cost of a drop in Precision to 93.6%, and its mAP50 failed to surpass the baseline model, indicating a suboptimal balance between precision and recall.

Comprehensive analysis of all metrics reveals that the proposed BiMAFPN exhibits the best overall performance. It not only maintains Precision at 94.1% but also elevates Recall to 78.7%, effectively compensating for the potential missed detection shortcomings of previous improvement modules. This advantage is directly reflected in the comprehensive evaluation metrics, where BiMAFPN achieved 83.5% in mAP50 and 64.5% in mAP50-95, both being the highest among all comparison groups. This fully proves that BiMAFPN, by combining bidirectional cross-scale connections with multi-scale attention mechanisms, successfully realizes efficient complementarity between deep semantic and shallow detail features, providing strong feature support for high-precision detection in complex scenarios.

#### 3.2.4 Comparison experiments among different models

To comprehensively evaluate the combat performance of CF-DETR, this study selected current mainstream detection models for horizontal comparison, including representatives of the Faster RCNN and DEIM Transformer architecture (RTDETR-r18, r34, r50) and the CNN architecture (YOLOv8, YOLOv13, YOLOv26). All models were trained under a unified dataset and hardware environment, with results shown in **Table 7**.

**Table 7:** Comparison of training results of different detection algorithms.

Model	P/(%)	R/(%)	mAP50/(%)	mAP50-95/(%)	FLOPs(G)	Params(M)	FPS(f/s)
Faster R-CNN	38.4	71.2	57.7	34.7	250.2	28.3	19.3
DEIM_hgnetv2 n	89.7	76.1	89.7	68.2	7.1	3.7	123.9
DEIM_rtdetrv2-r18	92.4	81.9	88.4	65.2	29.8	13.52	76.4
RTDETR-r18	93.2	78.5	81.5	62.4	56.9	19.8	80.0
RTDETR-r34	93.9	78.3	82.1	62.8	88.8	31.1	57.3
RTDETR-r50	91.4	79.8	83.3	63.2	129.5	41.9	42.7
YOLOv8n	93.7	74.4	81.5	61.4	8.1	3.01	134.8
YOLOv13n	90.6	76.6	82.2	60.9	6.2	2.45	46.6
YOLO26n	93.7	75.2	80.8	60.7	5.2	2.37	83.6
**CF-DETR**	**94.1**	**82.4**	83.5	64.5	62.0	20.1	56.3

Bold font means the best value.

A systematic evaluation of the comparison experimental results reveals more evident performance differences across various detection paradigms, especially after introducing two Faster R-CNN variants. As typical two-stage detectors, the Faster R-CNN models show clear limitations in this agricultural scenario. Their precision remains very low, reaching only 38.4% and 38.7%, while recall stays at 71.2% and 71.5%. The corresponding mAP_50_ results are also unsatisfactory, with values of 57.7% and 58.1%. These results indicate that such methods struggle to adapt to small, dense, and occluded pepper flower targets, mainly due to the inherent limitations of region proposal mechanisms in capturing fine-grained features under complex field conditions.

The two DEIM-based models provide a noticeable improvement over conventional detectors. DEIMrtdetrv2-n achieves a precision of 89.7% and a recall of 76.1%, while DEIM-rtdetrv2-r18 further improves the performance to 92.4% in precision and 81.9% in recall. Their mAP_50_ results reach 89.7% and 88.4%, and mAP_50–95_ reaches 68.2% and 65.2%, respectively. These results indicate that the DEIM mechanism enhances feature representation and improves the detection of dense targets. However, the increase in model capacity also leads to higher computational cost, especially for the r18 version, which shows a clear growth in parameters and FLOPs.

The YOLO series presents a different pattern. These models maintain relatively high precision ranging from 90.6% to 93.7%. However, their recall is limited between 74.4% and 76.6%, which leads to only moderate performance in mAP_50-95_, remaining around 60% to 61%. This suggests that although one-stage detectors are efficient and capable of fast localization, they are more likely to miss targets in dense and occluded environments.

The RTDETR models improve this situation by introducing global self-attention mechanisms. Among them, RTDETR-R50 achieves the highest recall of 79.8% while keeping competitive precision. Even so, its overall detection accuracy is still constrained, and mAP_50–95_ reaches 63.2%. In addition, this improvement comes with a noticeable increase in computational cost.

Based on these observations, CF-DETR shows clear advantages across all evaluation metrics. It achieves the highest precision of 94.1% and recall of 82.4%, demonstrating stronger capability in both classification and detection completeness. At the same time, it reaches 83.5% in mAP_50_ and 64.5% in mAP_50-95_, exceeding the baseline RTDETR-R18 by 2.1 percentage points in mAP_50_ and outperforming all other models. These improvements reflect its stronger feature representation and better robustness when dealing with small and densely distributed targets.

From the perspective of computational cost, CF-DETR also shows a good balance. Faster R-CNN and RTDETR-R50 require very high computation, reaching 250.2 GFLOPs and 129.5 GFLOPs. In comparison, CF-DETR keeps the complexity at 62.0 GFLOPs with 20.1 million parameters. At the same time, it achieves an inference speed of 56.3 frames per second, which meets real-time requirements. Although YOLO models can reach higher speeds up to 134.8 frames per second, this advantage is obtained at the cost of reduced detection accuracy.

Overall, CF-DETR provides a better balance between accuracy and efficiency. It effectively overcomes the limitations of both traditional two-stage detectors and lightweight one-stage models. With the integration of the FasterNet backbone, DySample upsampling, and BiMAFPN feature fusion, the model can extract multi-scale features more effectively and achieve more precise localization. This makes it well suited for practical agricultural applications such as automated monitoring of pepper flower maturity.

### 3.3 Comparison of visualization detection results of each model

**Figure 8:**
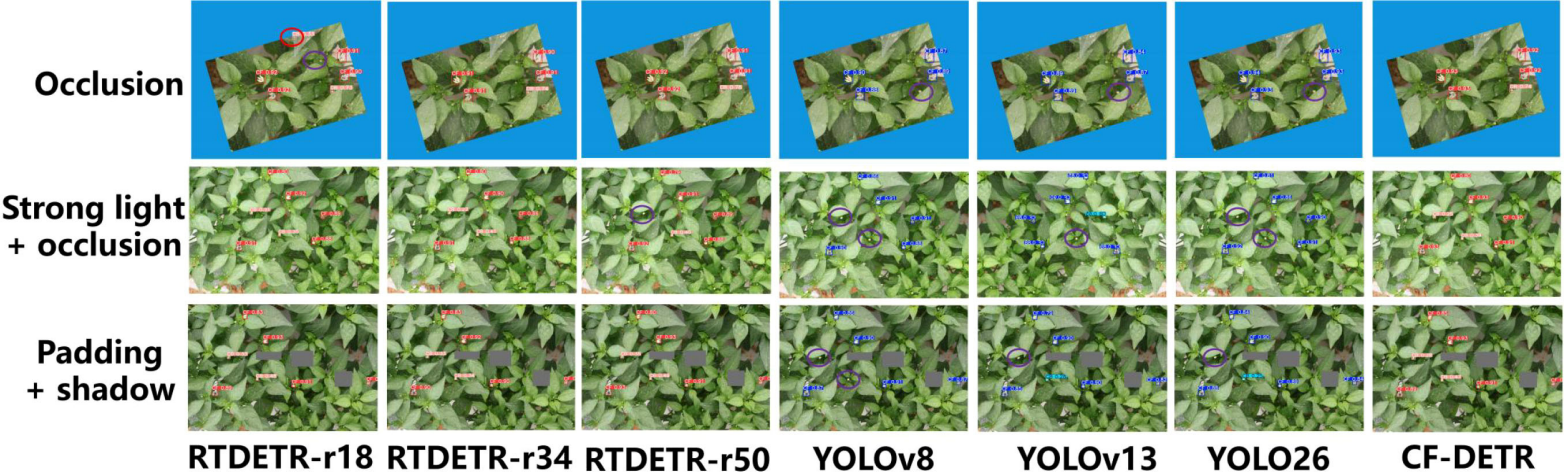
Comparison of visual detection results among different models under various environmental interferences including blur, strong light, and shadow, involving RT-DETR series, YOLO series, and the proposed CF-DETR.

**Figure 9:**
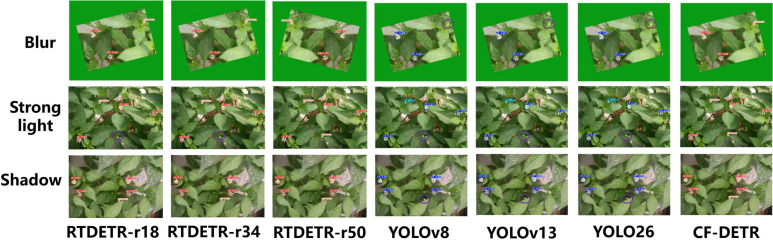
Comparison of visual detection performance among different models under various occlusion conditions, including simple occlusion, strong light with occlusion, and filling with shadow.

To comprehensively evaluate the combat performance of CF-DETR, this study selected current mainstream detection models for horizontal comparison, including representatives of the Transformer architecture (RTDETR-r18, r34, r50) and the CNN architecture (YOLOv8, YOLOv13, YOLOv26). **Figures 8**, **9** display the detection results of each model under extreme conditions such as blurring, strong light, shadows, and occlusion.

Firstly, analyzing the RTDETR series models as the baseline, significant architectural limitations were found when processing tiny pepper flower targets. As shown in **Figure 8**, the lightweight RTDETR-r18 exhibited a high missed detection rate in shadow scenarios, primarily due to the loss of key shallow texture information by its backbone network during downsampling. Although RTDETR-r34 improved feature extraction capabilities by increasing network depth, it did not show performance improvements commensurate with the increase in parameters in shadow scenarios, and corresponding false detections also occurred. Conversely, the excessive abstraction of semantic information brought by deep networks led to a dulling of the model’s perception of shallow tiny geometric features, making it still difficult to accurately identify tiny flower buds heavily occluded by leaves. This phenomenon indicates that merely stacking network depth cannot effectively solve the perception problem of tiny targets in unstructured environments and may instead introduce computational redundancy. Meanwhile, YOLOv8, YOLOv13, and YOLOv26 all exhibited false detections under blurred, strong light, and shadow scenarios, indicating that the robustness of their CNN architectures in dense and dynamic scenes is clearly insufficient.

Secondly, regarding the YOLO series models (v8, v13, v26), although they perform adequately under standard illumination, their robustness in dense and dynamic scenarios is notably insufficient. In the occluded scenarios depicted in **Figure 8**, the RTDETR-r18 model exhibited false positives by misidentifying leaf reflections as flowers, while YOLOv8 and YOLOv13 suffered from missed detections of tiny pepper flower targets. This limitation is primarily constrained by their reliance on the Non-Maximum Suppression (NMS) post-processing mechanism. When discerning small targets with high degrees of overlap, NMS is prone to erroneously suppressing candidate bounding boxes of genuine targets. Furthermore, despite the improvements in feature extraction within the lightweight YOLOv26, its missed detection rate in the occluded scenarios of **Figure 9** remained universally higher than that of CF-DETR. This indicates that the feature robustness of traditional CNN architectures still requires enhancement when handling image degradation and low signal-to-noise ratio conditions.

In contrast, CF-DETR, through targeted architectural innovations, demonstrated optimal comprehensive performance across all test scenarios. Benefiting from the inherent NMS-free characteristic of the RTDETR architecture, CF-DETR completely eliminated candidate box conflict issues in dense flower cluster detection, clearly defining the boundaries of each bud even in multi-target overlapping regions, as shown in **Figure 9**. Simultaneously, addressing small targets and multi-scale adaptation, the introduced BiMAFPN structure strengthens the fusion of deep semantics and shallow details via bidirectional cross-scale connections. When combined with the acute perception of spatial textures by the FasterNet backbone, the model enables the precise capture of tiny buds that are only minimally exposed in shadow and strong light scenarios. Additionally, in blur tests, thanks to the point-sampling mechanism of the DySample dynamic upsampling operator, CF-DETR effectively suppressed feature noise caused by image blurring, maintaining an exceptionally high Recall rate under low-definition conditions. In summary, CF-DETR effectively overcomes the shortcomings of existing mainstream models in “tiny, dense, and interference-prone” scenarios, proving its superiority as the preferred algorithm for greenhouse pepper flowering monitoring.

**Figure 10:**
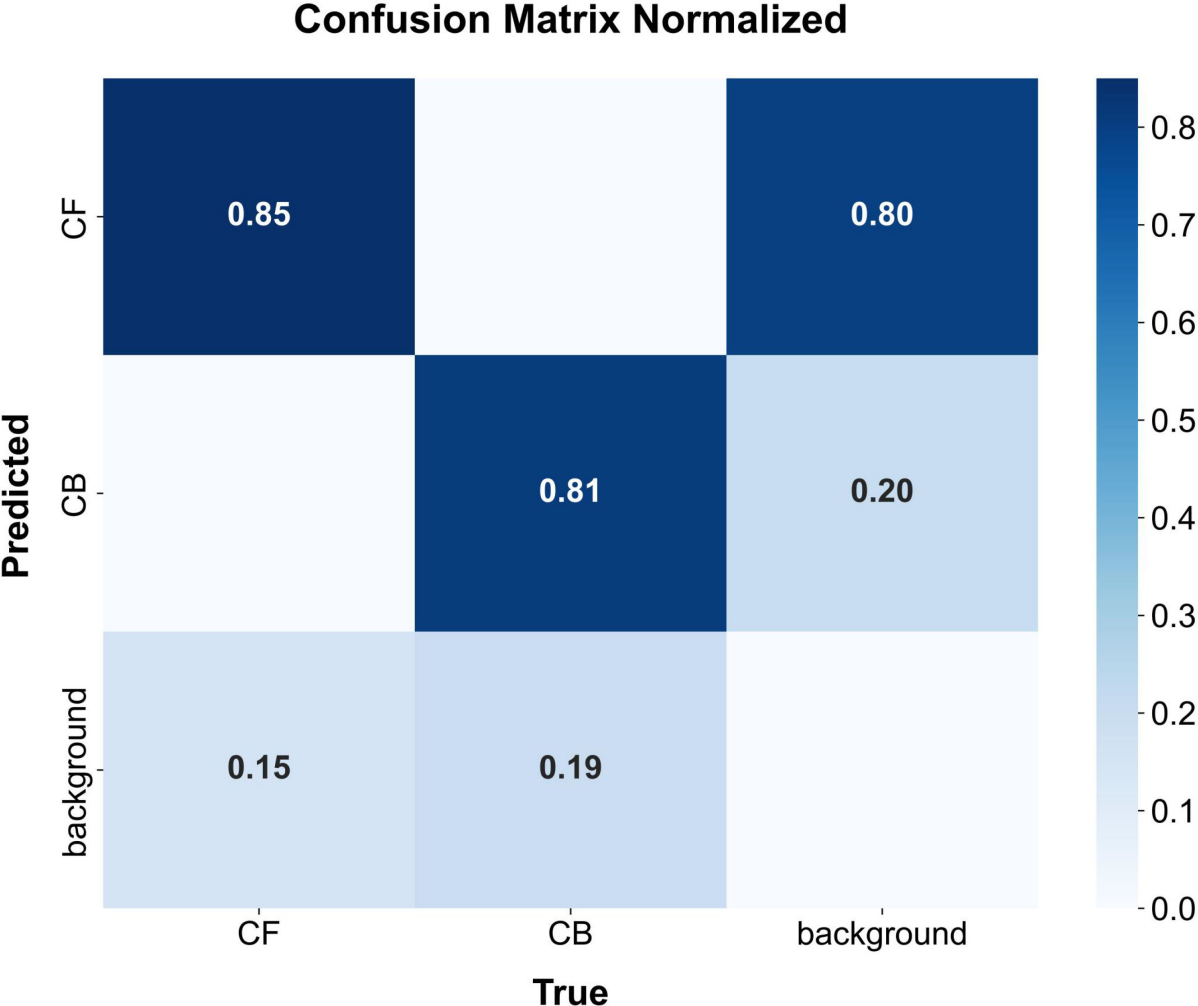
Normalized confusion matrix for chili flowering stage classification. The matrix quantifies the discriminative power of the CF-DETR model for three categories: Chili Flower (CF), Chili Bud (CB), and Background. The diagonal values represent the classification accuracy, with 0.85 and 0.81 achieved for flowers and buds, respectively. The low off-diagonal values indicate that the model effectively minimizes semantic confusion between morphologically similar developmental stages, though a small percentage of errors stem from background interference (e.g., specular reflections from foliage).

To further quantify the classification reliability of CF-DETR across different flowering stages, a normalized confusion matrix was generated for in-depth analysis (**Figure 10**). The results indicate that the model achieves high discriminative accuracy, with 85% for chili flowers (CF) and 81% for buds (CB), respectively. This performance underscores the superior inter-class discriminative power afforded by the integration of the FasterNet backbone and the BiMAFPN architecture. Specifically, the framework effectively captures subtle phenotypic cues, such as petal expansion and anther texture, successfully resolving the semantic ambiguity arising from the morphological similarities of reproductive organs during early developmental stages.

Analysis of the off-diagonal elements in the confusion matrix elucidates the impact of unstructured field environments on detection robustness. The false negative rates for CF (15%) and CB (19%) are primarily attributed to the severe foliar occlusion and the inherent feature sparsity of centimeter-level targets as previously discussed. Notably, the misclassification of background elements as flowers (0.80) highlights the significant perceptual challenge posed by specular reflections from leaves, whose textural signatures closely mimic those of white petals. Nevertheless, compared to conventional YOLO-series models where Non-Maximum Suppression (NMS) often triggers erroneous box filtering, CF-DETR leverages its NMS-free paradigm and DySample dynamic sampling operator to maintain high feature saliency, thereby optimizing the trade-off between precision and recall in cluttered agricultural scenes.

### 3.4 Chili flower heat map detection image comparison

**Figure 11:**
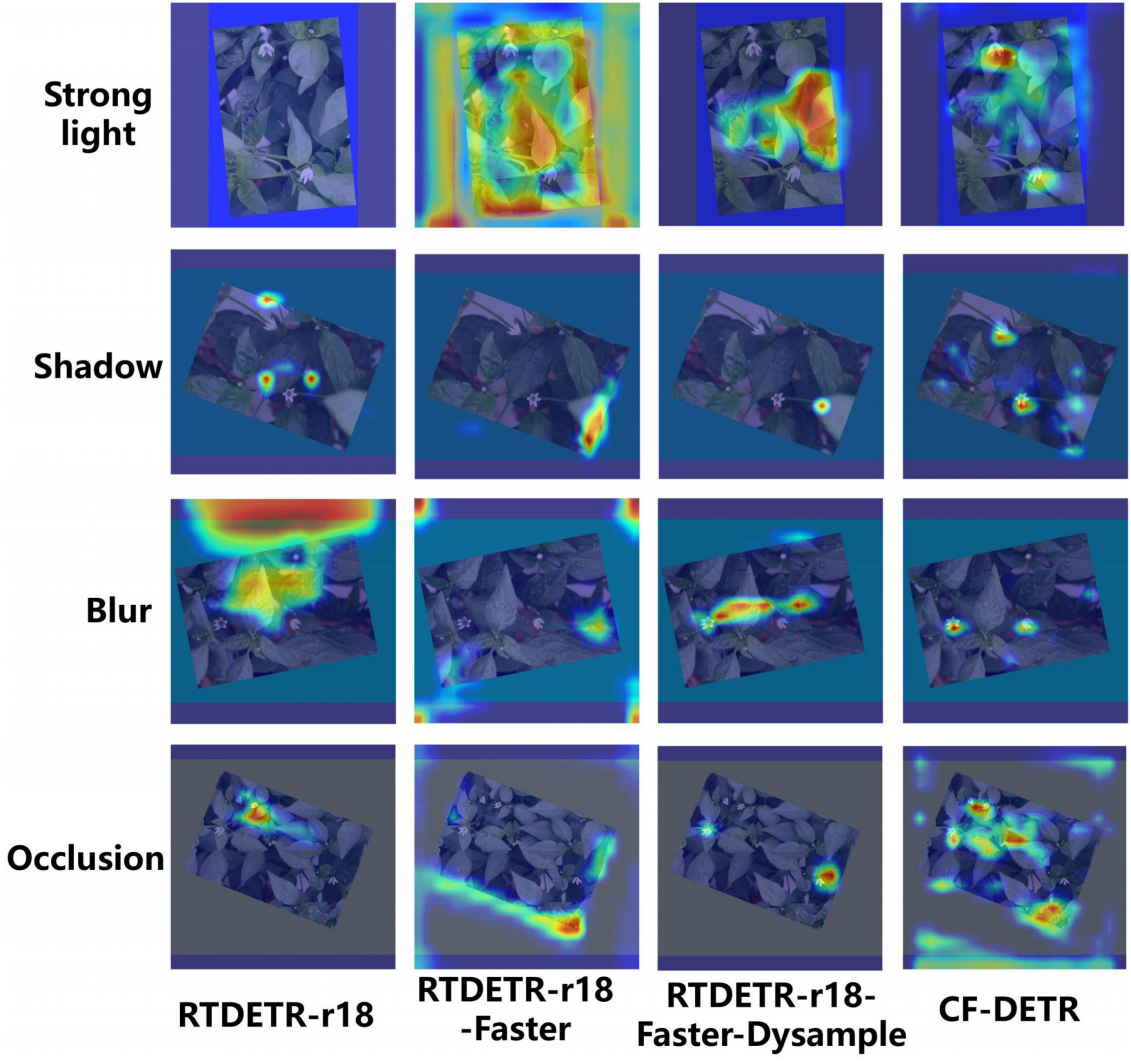
Comparison of heatmap-based visual detection results among different models under various scenes. The heatmaps are generated by Grad-CAM to show the attention regions of models under strong light, shadow, blur, and occlusion.

To intuitively analyze the decision-making mechanism and detection robustness of the models in complex unstructured environments, this study utilized Grad-CAM technology to generate heatmaps for models at different stages under varying lighting (strong light, shadow, blur) and occlusion conditions, as shown in **Figure 11**.

First, observing the visualization results of the baseline model RTDETR-r18 (first column), its thermal distribution exhibits a significant phenomenon of “attention divergence.” Particularly under strong light and blur interference, substantial high-activation red regions erroneously permeate the background foliage and ground weeds, whereas the thermal response targeting the pepper flower subjects is weak and discontinuous. This indicates that the original model is highly susceptible to induction by high-frequency background noise, making it difficult to effectively extract key discriminative features of tiny targets, leading to frequent false and missed detections in complex scenarios.

With the progressive introduction of improvement modules, the feature focusing capability of the intermediate-stage models was corrected to varying degrees. For the RTDETR-r18-Faster model (second column), after incorporating the FasterNet backbone, benefiting from PConv’s filtering of redundant features, its background false response in shadow scenarios was significantly reduced, and the thermal range began to contract toward the target region, although the edge contours remained relatively blurred. The RTDETR-r18-Faster-Dysample model (third column), which further integrates the DySample module, significantly improved the upsampling quality of feature maps. As seen in the figure, this model captured the edge textures of petals more acutely under blur and occlusion conditions, with a qualitative improvement in the aggregation of highlight regions compared to preceding models. However, in extremely dense occlusion areas, its ability to suppress interfering features appeared insufficient, with some thermal points still drifting away from the target subject.

The finally proposed CF-DETR model (fourth column) demonstrated optimal visual interpretability and environmental adaptability. Benefiting from the synergistic enhancement of the BiMAFPN feature fusion structure and prior improvements, the attention map generated by this model presents an ideal distribution of “target-hot, background-cold.” Whether in high-contrast strong light environments or under severe foliage occlusion, CF-DETR precisely locked the thermal focus onto the pistil and petal core regions of the pepper flowers. The generated activation regions possessed clear and compact boundaries, achieving near-perfect suppression of the surrounding complex foliage background. This result powerfully confirms that the CF-DETR model not only possesses strong anti-interference capabilities but also deeply mines the deep semantic information of tiny targets, thereby achieving precise localization in complex agricultural environments.

### 3.5 Deployment experiments

**Figure 12:**
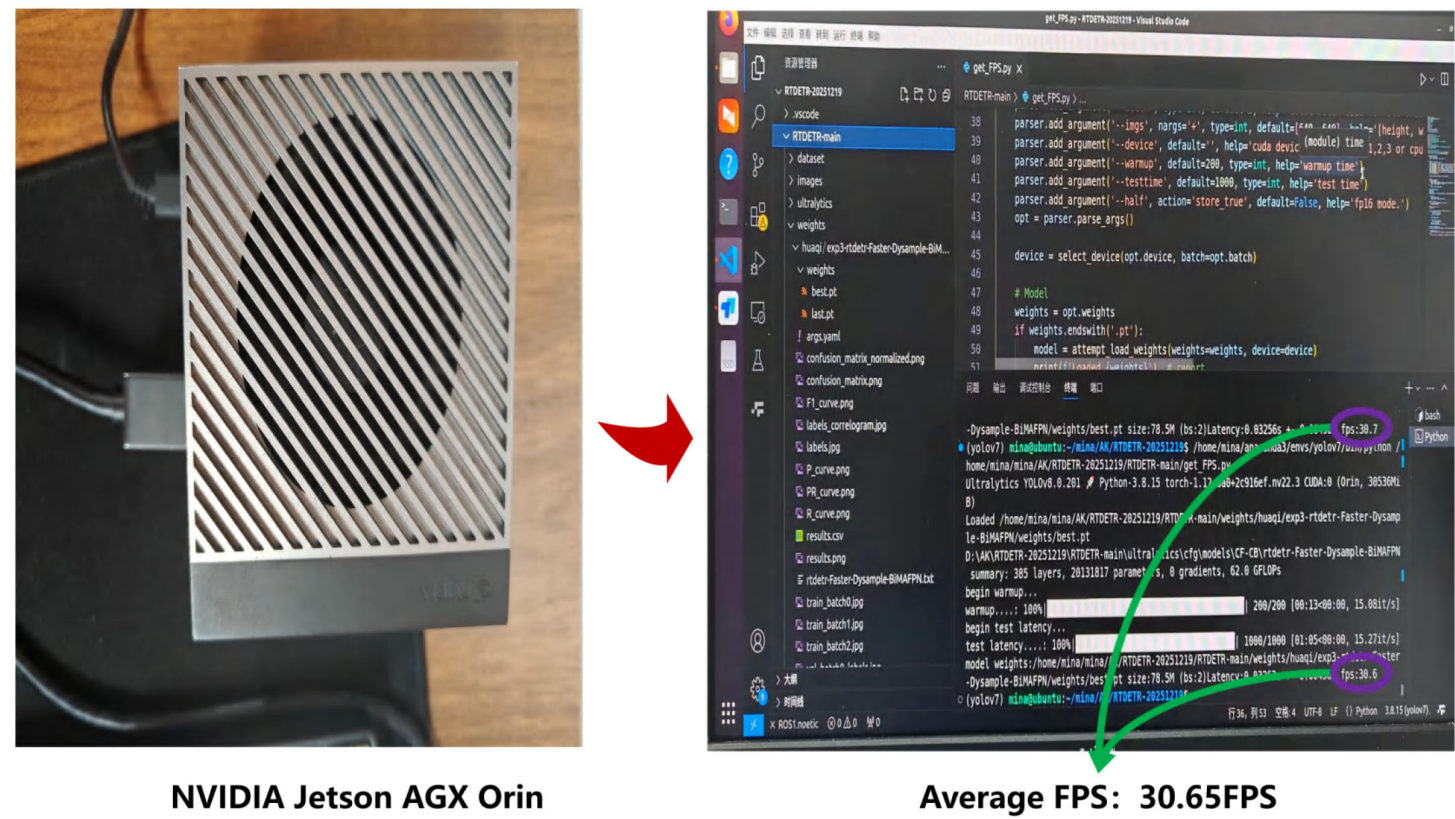
Deployment experiment on the NVIDIA Jetson edge computing platform, showing the NVIDIA Jetson AGX Orin hardware and the inference speed (FPS) screenshot during real-time running.

Given the stringent constraints on computational resources and power consumption inherent to onboard embedded devices in intelligent agricultural pollination, validating the algorithm’s operational efficiency on edge hardware is critical. To this end, moving beyond simulations on high-performance PC workstations, this study deployed the trained CF-DETR (Note: standardized from DCFP-YOLO based on context) model directly onto the NVIDIA Jetson AGX Orin embedded computing platform (as illustrated in **Figure 12**) to simulate a realistic robotic inference environment.

The real-time execution logs from the deployment test demonstrate the model’s stable inference capabilities on the edge device. Without compromising detection accuracy, the model achieved an average inference speed of 30.65 FPS. This corresponds to a processing latency of approximately 33 ms per frame, which meets the fluidity threshold for real-time video processing (30 FPS) and satisfies the real-time visual feedback requirements for pollination robots in dynamic environments. These results conclusively validate the effectiveness of the lightweight architectural design, indicating that the model can operate independently of heavy external computing units. It provides reliable visual perception support for pollination operations on resource-constrained embedded platforms, demonstrating significant engineering application value.

## 4 Discussion

The experimental results demonstrate that CF-DETR effectively improves the robustness of chili flowering detection under dense and occluded greenhouse conditions. Compared with the baseline RT-DETR-r18 and representative YOLO-based detectors, the proposed framework achieves more stable perception in scenarios characterized by clustered buds, overlapping flowers, and complex foliage backgrounds. From an industrial production perspective, this improvement is particularly significant because accurate flower counting and spatial distribution analysis directly influence pollination management, yield estimation, and production scheduling in large-scale greenhouse systems.

The performance enhancement is closely related to the architectural adaptations tailored to chili phenological characteristics. The redesigned backbone strengthens fine-grained texture extraction for minute buds, while the optimized multi-scale fusion mechanism improves feature consistency across different growth stages. More importantly, the NMS-free detection paradigm avoids suppression conflicts in densely clustered regions, which is critical for agricultural monitoring tasks that depend on reliable counting rather than merely bounding-box localization accuracy.

**Figure 13:**
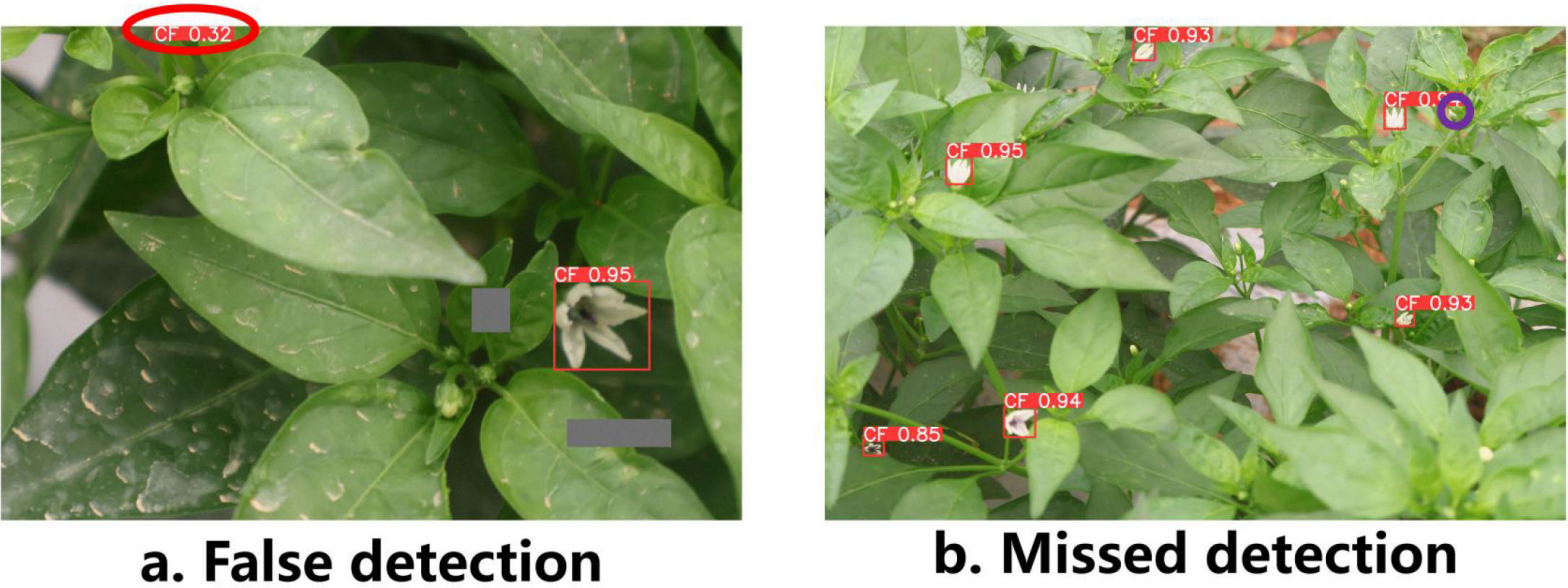
Analysis of false detection and missed detection cases, where **(a)** shows false detection examples and **(b)** shows missed detection examples.

Despite the overall improvement, analysis of failure cases (**Figure 13**) reveals several limitations under extreme environmental conditions. As illustrated in **Figure 13a**, false detections mainly occur in areas where strong specular reflections on leaf surfaces generate bright regions with similar chromatic characteristics to flower buds. In addition, edges of senescent or curled leaves sometimes produce contour patterns that resemble early-stage buds, leading to misclassification. These phenomena indicate that RGB-based perception may still be sensitive to reflectance artifacts and morphological ambiguity in high-humidity greenhouse environments.

Conversely, missed detections, shown in **Figure 13b**, are primarily associated with severe occlusion or deep shadow regions. When the occlusion rate exceeds approximately 80%, only a minimal portion of the bud remains visible, resulting in extremely weak visual signals that challenge the feature extraction network. Similarly, under low illumination or canopy shading, the contrast between buds and background decreases substantially, reducing discriminative feature strength. From a production standpoint, such missed detections may lead to underestimation of flowering density, which could influence pollination timing decisions or early yield predictions.

These observations suggest that while CF-DETR performs reliably under typical greenhouse operating conditions, robustness against extreme lighting variation and heavy occlusion remains a direction for further optimization. Integrating complementary sensing modalities or incorporating temporal continuity information may help mitigate the limitations identified in **Figure 13**. Nevertheless, under standard cultivation environments, the proposed framework demonstrates sufficient stability to support practical phenological monitoring in industrial chili production systems.

## 5 Conclusion and future works

This study presented CF-DETR, a chili flowering detection framework tailored for dense and small-target reproductive monitoring in greenhouse production systems. By redesigning the backbone architecture and optimizing multi-scale feature interaction, the proposed model achieves robust detection performance while maintaining real-time inference capability suitable for on-site deployment. Experimental results confirm that CF-DETR outperforms the baseline model in both precision and mAP metrics, and edge-platform validation demonstrates its feasibility for practical greenhouse applications.

Beyond detection accuracy, the primary contribution of this work lies in its relevance to industrial chili cultivation. Reliable flowering monitoring forms the foundation for intelligent pollination management, early yield prediction, and data-driven production regulation. By improving the stability of reproductive stage perception, the proposed framework supports more accurate estimation of potential fruit set and reduces uncertainty in industrial raw material supply. This contributes to enhancing production efficiency and strengthening agro-industrial value chains associated with chili processing industries.

Future research will focus on expanding both perception robustness and system-level integration. Multi-modal sensing approaches, such as RGB–depth or multi-spectral fusion, will be explored to mitigate visual ambiguity under extreme lighting conditions. In addition, temporal modeling of flowering dynamics across consecutive frames will be investigated to enhance detection continuity and resilience against occlusion. Furthermore, integrating flowering perception outputs with predictive yield models will be pursued to establish a stronger quantitative linkage between phenological monitoring and industrial production forecasting.

Through these developments, the proposed framework is expected to evolve from a high-performance detection model into a core phenotyping component within digitalized industrial horticultural production systems, contributing to the sustainable and intelligent transformation of chili cultivation.

## Data Availability

The raw data supporting the conclusions of this article will be made available by the authors, without undue reservation.

## References

[B1] Aquino A. Millan B. Diago M. P. Tardaguila J. (2018). Automated early yield prediction in vineyards from on-the-go image acquisition. Comput. Electron. Agric. 144, 26–31. doi:10.1016/j.compag.2017.11.026. PMID: 38826717

[B2] Bai Y. Yu J. Ning J. (2024). An improved YOLO algorithm for detecting flowers and fruits on strawberry seedlings. Biosyst. Eng. 237, 158–172. doi:10.1016/j.biosystemseng.2023.11.008. PMID: 38826717

[B3] Barik S. Ponnam N. Reddy A. C. Dc L. R. Saha K. Gc A. . (2022). Breeding peppers for industrial uses: Progress and prospects. Ind. Crops Prod. 178, 114626. doi:10.1016/j.indcrop.2022.114626. PMID: 38826717

[B4] Cao L. Li S. Jiang D. Sun M. Liu X. (2025). Egf-former: An efficient network for structural segmentation and phenotype extraction of sweet peppers in complex environments. Ind. Crops Prod. 227, 120850. doi:10.1016/j.indcrop.2025.120850. PMID: 38826717

[B5] Chen S. Zou X. Zhou X. Xiang Y. Wu M. (2023). Study on fusion clustering and improved yolov5 algorithm based on multiple occlusion of camellia oleifera fruit. Comput. Electron. Agric. 206, 107706. doi:10.1016/j.compag.2023.107706. PMID: 38826717

[B6] Govindarajan V. S. Salzer U. J. (1985). Capsicum-production, technology, chemistry, and quality part 1: History, botany, cultivation, and primary processing. Crit. Rev. Food Sci. Nutr. 22, 109–176. doi:10.1080/10408398509527412. PMID: 3899517

[B7] Guo L. Fu L. Wang X. Cui Y. (2022a). Multi-class detection of kiwifruit flower and its distribution identification in orchard based on YOLOv5l and euclidean distance. Comput. Electron. Agric. 201, 107313. doi:10.1016/j.compag.2022.107342. PMID: 38826717

[B8] Guo L. Suo R. Wang X. Cui Y. (2022b). Real-time detection of kiwifruit flower and bud simultaneously in orchard using YOLOv4 for robotic pollination. Comput. Electron. Agric. 193, 106693. doi:10.1016/j.compag.2021.106641. PMID: 38826717

[B9] Houtman W. Siagkris-Lekkos A. van de Molengraft M. J. G. (2021). Automated flower counting from partial detections: Multiple hypothesis tracking with a connected-flower plant model. Comput. Electron. Agric. 188, 106318. doi:10.1016/j.compag.2021.106346. PMID: 38826717

[B10] Kuang M. Li X. Chen S. Liu D. Xiang Y. Liu F. . (2025). Lightweight chili flower target detection method based on improved YOLOv8n. Trans. Chin. Soc. Agric. Eng. (Transactions CSAE) 41, 198–207. doi: 10.11975/j.issn.1002-6819.202502114

[B11] Kuang M. Li X. Xie F. Zou X. Xiang Y. Zhang Y. . (2026a). Advancements and prospects in key technologies for robotic pollination in greenhouse pepper breeding: a review. Front. Plant Sci. 17, 1778541. doi:10.3389/fpls.2026.1778541. PMID: 41835286 PMC12982380

[B12] Kuang M. Xie F. Li X. (2026b). A refined YOLOv5n-based method for detecting pepper flower objects integrating transfer learning. Appl. Soft Comput., 112852.

[B13] Kuang M. Zou X. Xie F. Li X. Chen S. Liu D. . (2026c). Ddm-yolo: A lightweight oriented detection model for mature daylily fruits in complex environments. J. King Saud Univ. Comput. Inf. Sci. doi:10.1007/s44443-026-00559-z. PMID: 30311153

[B14] Li X. Du J. Li Y. Li S. (2025). Deep learning-based kiwifruit flower recognition method to facilitate automated pollination. Appl. Soft Comput., 113063. doi:10.1016/j.asoc.2025.113855. PMID: 38826717

[B15] Li S. Li X. Li Y. (2021). Fast and accurate green pepper detection in complex backgrounds via an improved Yolov4-tiny model. Comput. Electron. Agric. 191, 106503. doi:10.1016/j.compag.2021.106503. PMID: 38826717

[B16] Li S. Li X. Whitty M. (2018). A robust automated flower estimation system for grape vines. Biosyst. Eng. 172, 35–46. doi:10.1016/j.biosystemseng.2018.05.009. PMID: 38826717

[B17] Li X. Liu Q. Kuang M. Wang S. Tang H. (2024). Detecting chili pepper fruits in a natural environment using improved YOLOX. Trans. Chin. Soc. Agric. Eng. (Transactions CSAE) 40, 119–126. doi: 10.11975/j.issn.1002-6819.202405175

[B18] Liang Z. Li X. Lin Z. Chen T. Liu B. Song M. . (2026). Pose estimation for long-staple cotton picking based on growth-relationship keypoint constraints and vision–language model reasoning. Ind. Crops Prod. 242, 122967. doi:10.1016/j.indcrop.2026.122967. PMID: 38826717

[B19] Liang Z. Li X. Wang G. Wu F. Zou X. (2025). Palm vision and servo control strategy of tomato picking robot based on global positioning. Comput. Electron. Agric. 237, 110668. doi:10.1016/j.compag.2025.110668. PMID: 38826717

[B20] Lu Y. Zhang Y. Liu H. Bader S. (2026). TinyIsn: A lightweight network for real-time marine pipeline leakage detection in IoT systems. IEEE Internet Things J., 1–1. doi:10.1109/jiot.2026.3665050. PMID: 25079929

[B21] Lyu Z. Wang Y. Shen Y. Zhao Z. (2024). Dynamic monitoring and counting for lotus flowers and seedpods with UAV based on improved YOLOv7-tiny. Comput. Electron. Agric. 225, 109313. doi:10.1016/j.compag.2024.109344. PMID: 38826717

[B22] Ma J. Wan Y. Min W. Wang Z. Zhang S. (2026). Frontiers and advances of deep learning-based fruit and vegetable image analysis. Comput. Electron. Agric. 241, 111256. doi:10.1016/j.compag.2025.111256. PMID: 38826717

[B23] Mendez E. Escobedo Cabello J. A. Gómez-Espinosa A. Cantoral-Ceballos J. A. Ochoa O. (2025). Capsicum counting algorithm using infrared imaging and yolo11. Agriculture 15, 2574. doi:10.3390/agriculture15242574. PMID: 30654563

[B24] Ollerton J. Erenler H. Edwards M. Crockett R. (2014). Extinctions of aculeate pollinators in britain and the role of large-scale agricultural changes. Science 346, 1360–1362. doi:10.1126/science.1257259. PMID: 25504719

[B25] Palacios F. Bueno G. Salido J. Diago M. P. Tardaguila J. (2020). Automated grapevine flower detection and quantification method based on computer vision and deep learning from on-the-go imaging using a mobile sensing platform under field conditions. Comput. Electron. Agric. 178, 105755. doi:10.1016/j.compag.2020.105796. PMID: 38826717

[B26] Shang B. Xie Q. Li N. Wang Y. Xu Y. (2023). Using lightweight deep learning algorithm for real-time detection of apple flowers in natural environments. Comput. Electron. Agric. 207, 107743. doi:10.1016/j.compag.2023.107765. PMID: 38826717

[B27] Shaowei M. Cheng L. Kui F. Xinghui Z. Chen G. (2026). Lgh-yolov12n: Latent diffusion inpainting data augmentation and improved yolov12n model for rice leaf disease detection. Agriculture 16, 368. doi:10.3390/agriculture16030368. PMID: 30654563

[B28] Sun K. Wang X. Liu Y. Liu C. (2021). Apple, peach, and pear flower detection using semantic segmentation network and shape constraint level set. Comput. Electron. Agric. 185, 106150. doi:10.1016/j.compag.2021.106150. PMID: 38826717

[B29] Tang Y. Qiu J. Zhang Y. Wu D. Cao Y. Zhao K. . (2023a). Optimization strategies of fruit detection to overcome the challenge of unstructured background in field orchard environment: A review. Precis. Agric. 24, 1183–1219. doi:10.1007/s11119-023-10009-9. PMID: 30311153

[B30] Tang Y. Zhou H. Wang H. Zhang Y. (2023b). Fruit detection and positioning technology for a camellia oleifera c. abel orchard based on improved yolov4-tiny model and binocular stereo vision. Expert Syst. Appl. 211, 118573. doi:10.1016/j.eswa.2022.118573. PMID: 38826717

[B31] Tian Y. Yang G. Liang Z. Wang X. Li E. Chen L. (2020). Instance segmentation of apple flowers using the improved mask R–CNN model. Biosyst. Eng. 193, 264–278. doi:10.1016/j.biosystemseng.2020.03.008. PMID: 38826717

[B32] Wang Z.-Y. Zhang C.-P. (2025). An improved chilli pepper flower detection approach based on YOLOv8. Plant Methods 21, 71. doi:10.1186/s13007-025-01390-9. PMID: 40426271 PMC12107810

[B33] Wu D. Lv S. Jiang M. Song H. (2020). Using channel pruning-based YOLO v4 deep learning algorithm for the real-time and accurate detection of apple flowers in natural environments. Comput. Electron. Agric. 178, 105742. doi:10.1016/j.compag.2020.105742. PMID: 38826717

[B34] Xu J. Zhang X. Zhang Y. Wang Y. (2026). ELSF-DETR: An efficient lightweight network for detecting strawberry flowers pollination status in non-structured greenhouse environments. Comput. Electron. Agric., 109852. doi:10.2139/ssrn.5404486

[B35] Xue Y. Li J. Chen T. (2026). Applications of image recognition in intelligent agricultural engineering: A comprehensive review. Agriculture 16, 496. doi:10.3390/agriculture16050496. PMID: 30654563

[B36] Ye J. Wu M. Qiu W. Yang J. Lan W. (2023). Litchi flower detection method based on polyphyletic loss function. Trans. Chin. Soc. For. Agric. Machinery 54, 235–242. doi: 10.6041/j.issn.1000-1298.2023.05.026

[B37] Zhang Y. Lu Y. Martinez-Rau L. S. Fan Z. Qiu Q. O’Flynn B. . (2026a). Tinyml-enabled iot edge framework with knowledge distillation for weed classification. IEEE Internet Things J., 1–1. doi:10.1109/jiot.2026.3679508. PMID: 25079929

[B38] Zhang Y. Lu Y. Martinez-Rau L. S. Qiu Q. Bader S. (2026b). Real-time on-device weed identification using a hardware-efficient lightweight cnn. Front. Plant Sci. 17, 1747863. doi:10.3389/fpls.2026.1747863. PMID: 41777389 PMC12950715

[B39] Zhang C. Mouton C. De Jong P. F. (2022). Automatic flower cluster estimation in apple orchards using aerial and ground based point clouds. Biosyst. Eng. 221, 1–16. doi:10.1016/j.biosystemseng.2022.05.004. PMID: 38826717

[B40] Zhang Y. Nurnberg A. Rau L. S. M. Vu Q. N. P. Lu Y. Oelmann B. . (2026c). Tinyml pipeline for efficient crack classification in uav-based structural health inspections. Sci. Rep. doi:10.1038/s41598-026-43534-4. PMID: 41813915 PMC12987979

[B41] Zhang Y. Zhang F. Wang J. Yang H. Zhang W. Li J. (2026d). Orchard chestnut visual harvest maturity detection and segmentation using an improved yolo-based method. Agriculture 16, 456. doi:10.3390/agriculture16040456. PMID: 30654563

[B42] Zhao C. Wen C. Lin S. Guo W. Long J. (2020). Recognition and detection method of tomato flowering period based on cascade convolutional neural networks. Trans. Chin. Soc. Agric. Eng. (Transactions CSAE) 36, 143–152. doi:10.12783/shm2017/13959. PMID: 41454284

[B43] Zhou X. Zou X. Meng H. Wu F. Chen S. Luo X. (2025). Multi-task perception and threedimensional picking point localization method for grapes based on structural constraints and geometric analysis. Comput. Electron. Agric. 238, 110814. doi:10.1016/j.compag.2025.110814. PMID: 38826717

